# Cellular Id1 inhibits hepatitis B virus transcription by interacting with the novel covalently closed circular DNA-binding protein E2F4

**DOI:** 10.7150/ijbs.62106

**Published:** 2022-01-01

**Authors:** Jie Wei, Yueyuan Shi, Chunhong Zou, Hongpeng Zhang, Hui Peng, Shilei Wang, Lulu Xia, Yuan Yang, Xiang Zhang, Junye Liu, Hua Zhou, Miao Luo, Ailong Huang, Deqiang Wang

**Affiliations:** 1 Key Laboratory of Molecular Biology for Infectious Diseases (Ministry of Education), Institute for Viral Hepatitis, Department of Infectious Diseases, The Second Affiliated Hospital, Chongqing Medical University, Chongqing, 400010, China.; 2 College of Laboratory Medicine, Chongqing Medical University, Yuzhong, Chongqing, 400016, China.; 3 Department of Clinical Laboratory, Zhuhai People's Hospital (Zhuhai hospital affiliated with Jinan University), Zhuhai, Guangdong, 519000, China.; 4 Division of Gastroenterology, Cedars-Sinai Medical Center, Los Angeles, California. Davis Bldg., Room 3094, 8700 Beverly Blvd., Los Angeles, CA 90048.; 5 Department of Clinical Laboratory, The People's Hospital of Yubei District of Chongqing City, Chongqing, 401120, China.; 6 Department of Laboratory Medicine, Chongqing Health Center for Women and Children, Chongqing, China, 401147, China.; 7 Department of Clinical Laboratory, The Second Affiliated Hospital of Chongqing Medical University, Yuzhong, Chongqing, 400010, China.

**Keywords:** HBV, E2F4, cccDNA, Id1, promoter.

## Abstract

Hepatitis B virus (HBV) infection is a major risk factor for hepatocellular carcinoma (HCC), which required developing novel therapies targeting the inhibition of HBV transcription and replication due to current limited treatment options. We explored novel target for the development of novel therapies targeting the inhibition of HBV replication and transcription. The expression of Id1 and E2F4 in HCC cells and tissues was detected by qRT-PCR and western blot. We investigated the Id1 and E2F4-mediated transcription of HBV infection by using HepG2.2.15, HepAD38, HepG2-NTCP cell lines and AAV/HBV-infected mice. Interactions between the two host proteins and viral covalently closed circular DNA (cccDNA) were assessed using subcellular localization, protein-protein interaction, chromatin immunoprecipitation, and luciferase assays. Ectopic Id1 significantly reduced HBV transcription and replication in both HBV-expressing cells and AAV/HBV-infected mice. Id1 and E2F4 could form a heterodimer to prevent E2F4 from promoting HBV transcription and replication. E2F4 could directly bind to cccDNA and activate the HBV core promoter in cell lines. Furthermore, *in vitro* binding experiments confirmed that the sequence 1758'-TTAAAGGTC-1766', which is highly conserved among HBV genotypes, is the target site of the E2F4 homodimer. The findings suggest that E2F4 function as novel cccDNA-binding protein to directly activate HBV transcription by binding to Cp promoter region. Our results highlight the ability that E2F4 represent a pan-potential therapeutic target against HBV transcription and provide more clues to better understand the life cycle of HBV.

## 1. Introduction

Despite notable improvements in the clinical treatment of hepatitis B virus (HBV) infection, up to 400 million individuals are chronically infected and at a high risk of developing primary liver cancer, with the majority of cases found in Asia and Africa 1,2. Moreover, there are still some shortcomings in treatments using pegylated interferon alpha (Peg-IFN-α) or nucleoside/nucleotide analogs, such as drug resistance and subsequent virological relapse 3-5. Therefore, novel and effective therapies are urgently required to inhibit HBV by targeting essential mechanisms of viral replication and transcription 6-9.

HBV belongs to the Hepadnaviridae family and exhibits a partially double-stranded relaxed circular DNA (rcDNA) genome. Once the sodium taurocholate co-transporting polypeptide (NTCP) mediates HBV entry into the hepatocyte, the viral rcDNA genome is transported into the nucleus and repaired into covalently closed circular DNA (cccDNA), which persists as episomal chromatin and serves as the template of transcription mediated by two enhancer regions, namely Enh 1 and 2, and four promoters, namely the core promoter (Cp), the surface promoter I (SPI), SPII, and the X promoter (Xp) 10. Subsequently, multiple cellular transcription factors, including HNF4α, NF-κB-P50, ZNF, VPS4b, and ZEB2, are recruited to the binding sites of the cccDNA minichromosome to regulate its transcription and, ultimately, viral replication 11-13.

So far, increasing host factors were found playing an important role in HBV infection, assembly, transcription and replication, but bHLH factors have not been found 14. Inhibitor of differentiation/DNA-binding 1 protein inhibitor (Id1), a liver-enriched factor, functions as a dominant negative inhibitor of basic helix-loop-helix (bHLH) transcription factors by blocking their binding to promoter DNA, and is highly expressed in HBV-related hepatocellular carcinoma (HCC) tumor tissues 15,16. However, total HBV DNA and RNA detected in TT samples were lowered than those in NT samples of HBV-related HCC patients (39% and 67% compared to 66% and 90%) 17. Id1 can interact with the HBV X (HBx) protein and target it for degradation in a proteasome-dependent manner and is negatively correlated with the stability of HBx 18,19. Hu et al. proposed that protein decline in HBV-infected cells is disadvantageous for HBV replication and vice versa; however, the detailed mechanism remains unclear 20.

E2F4, a member of the E2F family of transcription factors, can interact with pocket proteins such as p130, p107, and retinoblastoma protein (pRb), and recognize specific DNA sites found in a variety of promoters 21. E2F4 contains, but is not limited to, a DNA-binding domain (residues 1-86) and a dimerization domain (residues 86-180) that allows it to form heterodimers with dimerization partner proteins 22. The DNA-binding domain of E2F4, a highly conserved region in the E2F family, can bind to the core sequence motif 5'-TTTSSCGC-3' in promoter regions 23,24. E2Fs can be captured as DNA-binding proteins activating the adenoviral E2a promoter, implying that E2Fs are potentially involved in viral replication 25-27. E2F4 can enter the nucleus with the help of pocket proteins 28-30. However, whether E2Fs regulate HBV transcription and replication is not entirely clear. In the present study, we aimed to investigate the roles of Id1 and E2F4 in HBV replication and expression, as well as their interaction, thereby providing novel insights into antiviral drug design and development.

## 2. Materials and Methods

### 2.1 Plasmids

pCDNA3.1-HBV1.3 (HBV1.3) and pCDNA3.1-HBV1.1 (HBV1.1) plasmids, which respectively contained a HBV1.3-length and a HBV1.1-length transgene, pCMV-SPORT6/HBV1.1/HBc/HBp/HBs/HBx, Genetypes A/B/C/D HBV1.1 and HBV pGL3-Cp/Xp/sp1/sp2 were saved in our lab. Id1-shRNA1/2 and E2F4-shRNA1/2 plasmids were purchased from BIOTEND (China). Pmcherry-Id1, E2F4-pEGFP, Id1-pET28a and E2F4-pEGX-6p-1, which could express corresponding fusion-protein, were constructed in our lab. We constructed the E2F4*_1-180_*-pET28a plasmid, which expressed the 1-180 amino acid. Considering the E2F4 binding site motif, three candidate sites for E2F4 binding were predicted, including site 1: 1638'-GTTGCCC-1644', site 2: 1758'-TTAAAGGTC-1766', and site 3: 1798'-GTCTGCGC-1805'. Then, pGL3-Cp△site1, pGL3-Cp△site2 and pGL3-Cp△site3 were the mutants of plasmid pGL3-Cp.HBV1.3-mut, whose binding sites of E2F4 were mutated, were constructed from HBV1.3 vector (the primers for clone and site-specific mutagenesis were listed in the Supplementary Table S2. The sequences of siRNAs targeting E2F4 and Id1 were listed in Table S3).

### 2.2 Clinical samples

The study protocol was approved by the Clinical Research Ethics Committee of the Chongqing Medical University in January 2018 and was in compliance with the Declaration of Helsinki. All study participants provided written informed consent. Table S1 shows the characteristics of the patients in the present study, including four females and 21 males with a median age of 53.4 (22.0-77.0) years. Twenty-five liver tissues were obtained by liver biopsy. Proteins were directly extracted from tumor tissues (T1-25) and adjacent non-tumor tissues (N1-25) for western blotting.

### 2.3 Cell lines

HEK293, LO2, SMMC-771, HepG2, HepG2.2.15, and HepAD38 cells were purchased from BioVector NTCC (Beijing, China), and HepG2-NTCP cells were generously donated by Prof. Ninshao Xia (Xiamen University, China). All cells were incubated at 37℃ in a thermostat with 5% CO_2_.

### 2.4 HBV infection in HepG2-NTCP cells

HBV virus particle collection and infection were conducted as described before 31. Briefly, HepG2-NTCP cells were inoculated with HBV viral particles derived from supernatants of cultured HepAD38 cells at 400, 800, and 1600 genome equivalents (GE) per cell in the medium containing 4% PEG 8000 for 12 h, then the virus was removed from infected cells by washed with PBS three times, and the cells were maintained in Williams' E media before harvest.

### 2.5 HBeAg analysis

HBeAg was measured in mouse sera and culture supernatant (1:5 dilution) using Detection Kit (KHB, China) by a validated two-step immunoassay (chemiluminescent microparticle immunoassay) for the qualitative detection.

### 2.6 qRT-PCR

Cells were seeded into the wells of a 6-well culture plate and allowed to grow until 60%-80% confluence. Subsequently, these cells were treated with corresponding plasmids, shRNA or Adenovirus for 36 hours, total RNA was isolated from the cells or liver tissues using TRIzol reagent (Invitrogen, USA). The RNA was reverse transcribed to cDNA using AMV-Reverse Transcriptase (Roche, Switzerland). qRT-PCR was performed for Id1, p16, p21, p53, NFԟB-P50/P65, ZNF, VPS4b, E2F1/3/4/8, ARNT, ARNT2, HIF1α, HNF4ɑ, HNF6ɑ, pgRNA, HBV DNA, HBV cccDNA, β-actin , GAPDH and CA2, et al. (The primers for qPCR were listed in the Supplementary table S4) using an BIORAD Sequence Detection System as well as the SYBR Green master mix (Promega, USA). Cycling conditions for amplification were 95℃ for 30sec; 35 cycles at 95℃ for 5sec, 60℃ for 15sec, and 60℃ for 30 sec; and, finally, melt curve were used for evaluating the specificity of these primers. In the same sample, each gene expression was normalized by either GAPDH or β-actin mRNA copies.

### 2.7 Western blotting

HCC cell lines in a well of 60×15mm Dish or tissue samples were lysed 300 μl RIPA Lysis Buffer (Beyotime, China) supplemented with 1mM Phenylmethanesulfonyl fluoride. The protein concentration was determined using the Bradford assay. 20 μg or 50 μg of total protein was electrophoretically separated on 12% SDS-PAGE gel and transferred from the gel onto a PVDF membrane (Millipore, USA). Western blotting was performed according to standard procedures. PVDF membranes were washed with TBST containing NaCl, Tris-HCl, and Tween-20 and incubated with primary antibodies against target proteins at 4 ℃ overnight, followed by five washes with TBST. PVDF membranes were incubated with the appropriate secondary antibodies at room temperature for 1-2 h and washed five times with TBST. Finally the membranes were visualized by chemiluminescence with an ECL kit (Biorad, USA). Antibodies against Id1, E2F4, GFP, Cherry, GST, His, GAPDH and β-actin obtained from Santa Cruz Biotechnology (USA). Rabbit anti-HBc poly-clonal antibody (B0586) was obtained from Dako (Denmark).

### 2.8 HBV DNA extraction and Southern blotting

The viral particles were precipitated using 200 μL 35% PEG8000 with 1.5M NaCl by centrifugation at 20,000 g for 5 min at 4 ℃ and subsequently digested using 20 μL proteinase K (1 mg/mL) in 450 μL buffer (10 mM Tris-HCl, 10 mM EDTA and 0.5% SDS, pH 8.0) overnight at 45 ℃. Finally, the HBV DNA was extracted twice with 450 μL phenol/chloroform extraction buffer, and precipitated with 320 μL isopropanol, and washed with 500 μL ethanol, dried and dissolved in 20 μL ddH_2_O, and finally separated on 0.9% agarose gels. Southern blot analysis of HBV DNA was performed using DIG DNA Labelling and Detection Kit (Roche, Switzerland) as per manufacturer's instructions.

### 2.9 HBV cccDNA extraction

Seven days after cell infected with Id1Ad or GFPAd, the cells were lysed with Hirt lysate (50mmol /L Tris-HCl, 10mmol /L EDTA, 1% SDS, 150mmol /L NaCl), and genomic DNA was precipitated by adding 5mol /L NaCl at 4℃ overnight. After centrifugation at 12 000g for 30min, the supernatant was extracted with phenol/chloroform and precipitated with ethanol. Linear dsDNA were digested with Plasmid-Safe^TM^ ATP-dependent DNase (Epicentre, USA), but cccDNA was reserved. The purified cccDNA was used for qPCR assay with CA2 (Cyclin A2) as internal reference gene.

### 2.10 Immunofluorescent assay

The HepG2.2.15 cells were grown in six well plates with cover slips. After 48 hours, cells were fixed with 4% paraformaldehyde for 15 min followed by incubation PBST with 0.5% Triton X-100 for 20 min. Cells were blocked by goat serum and then incubated with Id1 and E2F4 antibodies at room temperature for 1 h. Mouse anti-Id1 monoclonal antibody (sc-133104) and mouse anti-E2F4 monoclonal antibody (sc-511) was obtained from Santa Cruz Biotechnology (USA). Bound primary antibody was visualized by Alexa Fluor 488-conjugated secondary antibodies and Alexa Fluor 647-conjugated secondary antibodies (Invitrogen, USA). Besides, Id1 and E2F4 nuclear localization were detected by immunofluorescent assay in HepG2.2.15 cells. Cell nucleuses were stained with DAPI. Cells were observed with a FluoView FV1000 laser scanning confocal microscope (Leica, Germany).

### 2.11 Immunoprecipitation

IP analysis was performed according to previously established methods. Cells were lysed with lysis buffer (50mM Tris-Cl pH7.5, 50mM NaCl, 5mM EDTA, 1% NP-40). The lysate containing 500 mg/ml cellular proteins was incubated with primary antibody (1:500; Abcam, Britain) overnight at 4℃. To detect the protein-protein interactions, co-immunoprecipitation (co-IP) was performed using protein A/G coupled to agarose beads (Millipore, USA) as per manufacturer's instructions. The protein A/G agarose beads were washed three times in PBST and then incubated with lysate mixture for 4 h at 4℃. Then beads were washed three times again in PBST. The immune complex was released from the beads by boiling in 2× SDS sample buffer. Finally, the target proteins were detected by western blotting.

### 2.12 GST Pull-down assay

To detect the Id1 protein interactions, the bacterial cell lysate of BL21 (DE3) expressing the protein of interest was allowed to bind to the Ni^2+^ affinity column. For *in vitro* pull down assay, the purified recombinant proteins GST-E2F4 used as bait was bound to GST affinity columns. Alternatively, His-Id1 protein was used as prey protein. The column was washed with at least 20 bed volumes of PBS. The bound proteins were eluted by glutathione and subjected to SDS-PAGE followed by western-blot analysis. The GST protein alone suffered the same treatment as GST-E2F4 was used as negative control to grab Id1 for verifying the specific binding between Id1 and E2F4.

### 2.13 Luciferase Reporter Assay

A luciferase reporter assay was performed to assess the effects of E2F4-induced HBV pGL3-Cp/Xp/sp1/sp2 activity, the effects of other transcription factor-induced HBV pGL3-Cp activity and the effects of E2F4-induced HBV pGL3-Cp mutant (including △site1-4). Plasmid pGL3-TK expressing the Renilla luciferase protein was used to normalize the transfection efficiency. Briefly, 8×10^4^ HEK293 cells per well were seeded in a 24-well plate 1 day before transfection. Four hundred nanograms of HBV pGL3-Cp/Xp/sp1/sp2 or HBV pGL3-Cp mutant with 5 ng of pGL3-TK were used for cell transfection with 1.2 μl of Lipofectamine 2000 (Thermo Fisher Scientific, USA) according to the manufacturer's instructions. Forty-eight hours after transfection, dual luciferase assays (Promega, USA) were performed using the cell lysates according to established protocols. The results were normalized for the internal Renilla luciferase control.

### 2.14 Electrophoretic Mobility Shift Assay EMSA

The primer sequence for HBV Cp double-stranded probes are listed in the Supplementary material Table S2. HBV Cp double-stranded probes were amplified by PCR. The binding activity of E2F4*_1-180_* to the probe was determined using a chemiluminescent Electrophoretic Mobility Shift Assay (EMSA) kit (Beyotime Biotechnology, China), according to the manufacturer's protocol. 4.5, 3.0 or 1.5 μg of E2F4*_1-180_* protein and 1 ng of probes was used in the binding reaction. The DNA-protein complex was electrophoretically separated on a 6% non-denaturing polyacrylamide gel. The gel was transferred to a nylon membrane and biotin was detected using streptavidin-based chemiluminescent enhanced chemiluminescence substrate. Specificity of the DNA-protein complex was confirmed by competition with a 10-fold molar excess of unlabeled double-stranded DNA added to the mixture. Bovine serum albumin replaced E2F4*_1-180_* protein as negative control.

### 2.15 Chromatin immunoprecipitation

A ChIP (chromatin immunoprecipitation) assay was performed to investigate whether Id1 influences the recruitment of E2F4 transcription factor to the candidate binding sequence (5'-TTGAGGC-3') in the HBV Cp in HepG2.2.15 and HepAD38 cells. Cells lysate was sonicated on ice. The lysates were centrifuged and the chromatin fraction in supernatant was subjected to immunoprecipitation with E2F4 antibody overnight at 4°C with non-specific immunoglobulins and the input as negative control and positive control, respectively. After elution and purification, the recovered DNA samples were used as a template for PCR (Takara, Japan) or qPCR, according to manufacturer's instructions. The primers for ChIP are listed in the Supplementary Table S4.

### 2.16 Construction of expression plasmid pET28-E2F4

The nucleotide binding domain of E2F4, assessed using ExPASy (https://www.expasy.org/), was predicted to consist in residues 1-180. According to the amino acid sequence of E2F4*_1-88_* and E2F4*_1-180_*, the codon was optimized for *E coli* expression system, and the optimized gene fragment (Table S5) was then synthesized, digested with *Nde*I and *Xho*I, and ligated to vector pET28a at 16 ℃ overnight. And the inserted DNA fragment was confirmed by DNA sequencing.

### 2.17 Soluble expression and purification of E2F4

The plasmids pET28-E2F4*_1-88_* and pET28-E2F4*_1-180_* was transformed *E. coli BL21* (DE3) and grown in LB medium at 37 ℃ until an optical density of 0.4-0.6 (OD600) was reached. Next, 0.1 mM IPTG (isopropyl-β-d-thiogalactoside) was added and the temperature was lowered to 20 ℃ and allowed to incubate overnight. Cells were then harvested, resuspended in ice-cold buffer A (20 mM Tris-HCl at pH 8.0 and 300 mM NaCl), and then lysed by sonication in ice-bath. The cell debris was removed by centrifugation and the resulting soluble fraction applied to Ni^2+_^NTA affinity resin (GE Healthcare) prebalanced by buffer A (10 mM imidazole, 10 mM sodium phosphate pH 7.4 and 300 mM NaCl). After washing (60 mM imidazole, 10 mM sodium phosphate pH 7.4 and 300 mM NaCl), the protein was eluted from the resin with buffer C (500 mM imidazole, 10 mM sodium phosphate pH 7.4 and 300 mM NaCl). The purity of the protein was estimated to be higher than 85-95% by SDS-PAGE. The puried protein was desalted and concentrated by an Amicon Centriprep YM-10 (Millipore).

### 2.18 Oligomeric state determination

For the soluble expression of E2F4*_1-180_* was much lower than that of E2F4_1-88,_ the purified E2F4*_1-88_* protein was determined next size exclusion chromatography. The protein sample or molecular mass standards were applied to the Superdex 75 column and eluted with 50 mM Tris-HCl pH8.0 and 300 mM NaCl. Protein molecular weight standards used were: bull serum albumin (67.0 kDa), chicken egg albumin (44.2 kDa), lactoalbumin (24.5 kDa), ribonuclease A (13 kDa); the void volume was determined with Blue Dextran (GE Healthcare). The peak elution volumes were used for the calculation of the standard curve equation (Log MW = -0.174Ve+6.5043, with the R2 value of 0.9848).

### 2.19 SPR binding assay

Biotinylated DNAs, which were synthesized from BGI (The Beijing Genomics Institute), were firstly dissolved at a final concentration of 1000 nM in 10mM phosphate buffer saline consisting of 137mM NaCl, 2.7mM KCl, 10mM Na_2_HPO4, 2mM KH_2_PO4 (pH 7.4). In advance, the biotinylated DNAs were fixed on streptavidin-coated sensor chips (Sensor chip SA and Biacore X100 both were obtained from GE Healthcare, USA) according to the instructions of the manufacturer. There were two flow cells in the sensor chips: one for HBV Cp-DNA being immobilized, while the other flow cell for reference Control-DNA. To measure binding off-rates of E2F4*_1-180_* protein and HBV Cp-DNA, we delivered E2F4*_1-180_* protein solutions (413 nM protein concentration) at a flow rate of 30 μl/min and with a contact time for the DNA sensor of 180 s, which was followed by a dissociation time of 90 s. Before next experiment, the sensor chips were injected with 2 M NaCl with 40 μl/min to remove the chip residual protein, E2F4*_1-180_*. According to the method of Thanos D. Halazonetis et al, the SPR RUs determined during the dissociation of E2F4*_1-180_* from the sensor chip were used to calculate off-rate constants. To further determine E2F4*_1-180_* DNA binding affinity constants, E2F4*_1-180_* protein solutions ranging from 10 to 320 nM were delivered at a flow rate of 10 μl/min and with a contact time for the DNA sensor of 180 s, followed by a dissociation time of 180 s. Between experiments, the sensor chips were rinsed by the same method as above indicated. The DNA fraction immobilized was determined as the ratio of the observed difference of RUs upon E2F4*_1-180_* binding (ΔRU-E2F4*_1-180_*) divided by the RUs corresponding to the theoretical maximum difference (ΔRU_maxθ_). The latter is equal to ΔRU_Cp-DNA_ × (MW-E2F4*_1-180_*/MW_Cp-DNA_) ×K, where ΔRU_Cp-DNA_ is the observed difference of RUs upon binding of DNA to the chip; MW_Cp-DNA_ and MW-E2F4*_1-180_* are the molecular weights of the DNA duplex and the E2F4*_1-180_* dimer respectively; and K means the fraction of DNA coated on the chip that is capable of binding to E2F4*_1-180_*. Experiments with increasing amounts of E2F4*_1-180_* revealed that K was equal to 0.5. Control-DNA used for the randomly reference DNA was subjected to the same process.

### 2.20 Isothermal titration calorimetry (ITC) Sample Preparation

The E2F4*_1-88_* protein mutant (1-264 bases, containing the structure domain of binding HBV Cp) was purified using GST affinity column, subsequently digested with 3C protease and dissolved in the buffer with 250 mM NaCl and 50 mM Tris-HCl, pH7.5. To generate the DNA duplex, the single-stranded oligonucleotide and its complementary oligonucleotide were incubated for 10 mins in the buffer from Centrifugal ultrafiltration concentration of E2F4*_1-88_* protein mutant at 100 ℃, and the temperature was slowly decreased to allow optimal annealing.

### 2.21 Animal studies

Adenovirus [a mixture of 5 × 109 GFU (green colony forming units) of Id1Ad, siE2F4Ad, and GFPAd ] was dissolved in 0.3 mL of saline and injected into male, 6-8 weeks old AAV/HBV-infected mice through the tail vein over a period of 10 s [ HBV-infected mice were produced by microinjection of pAAV/HBV1.2 (plasmid of adeno-associated virus containing 1.2 copies of HBV genome) into the embryos of C57BL/6 mice. The founders were screened by analyzing the serum HBsAg and HBeAg, and the one that produced highest level of HBsAg and HBeAg with active intrahepatic HBV replication (confirmed by Southern and northern hybridization) was chosen for the current study 32,33. AAV/HBV-infected mice were kindly provided by Prof. Ning-shao Xia from the School of Public Health, Xiamen University]. The serum and liver tissue were harvested 4 weeks after viral injection. All animal studies were performed in accordance with protocols approved by the Rules for Animal Experiments published by the Chinese Government and approved by the Research Ethics Committee of Chongqing Medical University (*reference number: 2019003*).

### 2.22 Statistical analysis

Data are presented as the mean ± standard error of the mean (SEM) of at least three independent experiments. All statistical analyses were performed using SPSS 19.0 and GraphPad Prism 8.4 software. Student's *t*-test was used to compare two groups. A value of *P* < 0.05 was regarded as statistically significant (*P < 0.05; **P < 0.01).

## 3. Results

### 3.1 Id1 is negatively correlated with HBV transcription and replication

**Figure 1:**
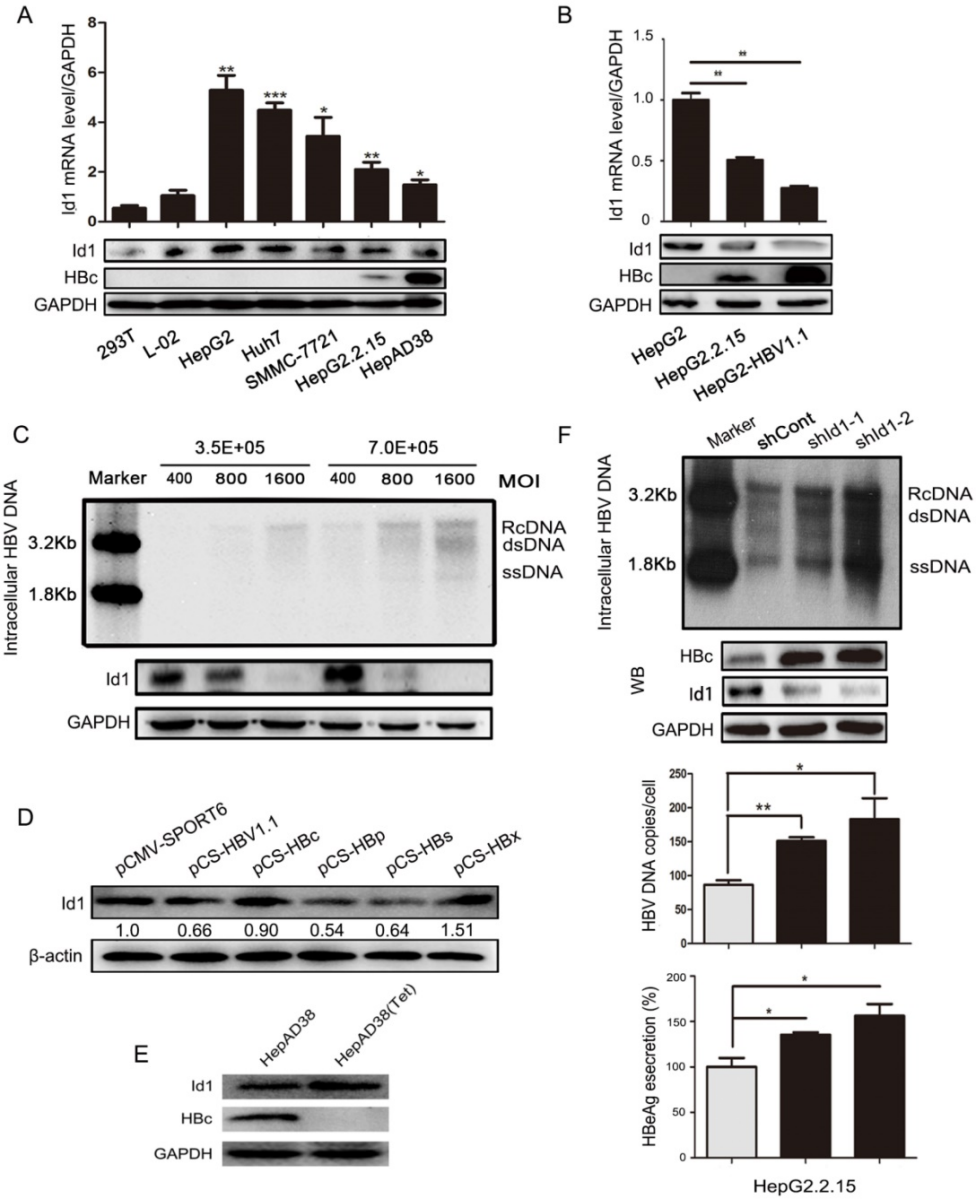
The comparison of Id1 expression in HBV or non-HBV context, and Id1 knockdown promoted HBV replication. **(A)** The Id1 mRNA (top layer) and protein (bottom layer) levels in 293T, L02,HepG2, Huh7,SMMC-7721,HepG2.2.15 and HepAD38 cells were analyzed by qPCR and western blotting with GAPDH used as the control gene. *P < 0.05, **P < 0.01 and ***P < 0.001. n = 5. **(B)** The Id1 mRNA (top layer) and protein (bottom layer) levels in HepG2, HepG2.2.15 and HepG2-HBV1.1, which was transiently transfected with plasmid HBV1.1, were detected by qPCR and western blotting with GAPDH used as the control gene. **P < 0.01. n = 5. **(C)** 3.5×10^5^ or 7×10^5^ Cells/35mm dish were infected with 400, 800 and 1600 HBV particles /cell. Then the cells were lysed and extracted for HBV DNA, which followed with southern blotting (top panel). Id1 protein levels of each cell dish infected with different virus titer of HBV were detected by western blotting (bottom panel). **(D)** Four plamids respectively expressing HBc/p/s/x proteins and HBV1.1 were transfected into HepG2 cells to verify the effect of HBV on Id1. **(E)** The HBc and Id1 protein level in HepAD38 cells with tetracycline-inhibiting HBV transcription was detected by western blotting with GAPDH used as the control gene. **(F)** HepG2.2.15 cells transfected with shRNA plasmids targeting Id1 or shCont vector as control group were harvested 72h, and intracellular HBV DNA was extracted and precipitated by isopropanol after viral infection**.** HBV DNA was determined by southern blotting and qPCR. Southern blotting analysis: Maker 1.8kb and 3.2kb; RcDNA, HBV relax circle DNA; dsDNA, double-stranded DNA; ssDNA, single-stranded DNA. The qPCR data were expressed as the number of HBV DNA copies per cell. HBc and Id1 protein was determined by Western blotting. Meanwhile, GAPDH was used as a loading control. Expression of HBeAg in the supernatant of cell culture media were detected using ELISA 3 days after transfection. *P < 0.05, **P < 0.01. n = 5.

Id1 is predominantly expressing in hepatocytes and critical for cell cycle and cell differentiation. We had previously demonstrated that HBV dramatically inhibit the expression of Id1 via the BMP/Smad signaling pathway and ubiquitination pathway mediated by HBc and HBx protein 19. So, in present study, we sought to determine effect of Id1 on HBV transcription and replication. Both the mRNA and protein levels of Id1 were significantly lower in HBV-expressing cells than in HBV-negative cells (Fig. 1A and 1B). Intriguingly, Id1 protein declined in a dose-dependent manner according to the infection load of the HBV particles in HepG2-NTCP cells (Fig. 1C) and decreased over time, even though HBV replication declined (Fig. S1A). HepG2 cells were transiently transfected with the four viral genes, and only *HBp* and *HBs* exhibited a remarkable inhibitory effect on Id1 expression (Fig. 1D). Furthermore, there were analogous trends of differential Id1 expression between AAV/HBV-infected mice and control mice (Fig. S1B). On the contrary, Id1 was upregulated in HepAD38 cells after adding tetracycline (Fig. 1E). Next, we investigated whether Id1 could influence viral replication and transcription by knockdown with short hairpin RNA targeting Id1 (shId1-1/2) in HepG2.2.15 cells, and we found this to be the case (Fig. 1F).

### 3.2 Overexpressing Id1 suppresses HBV transcription and replication

**Figure 2:**
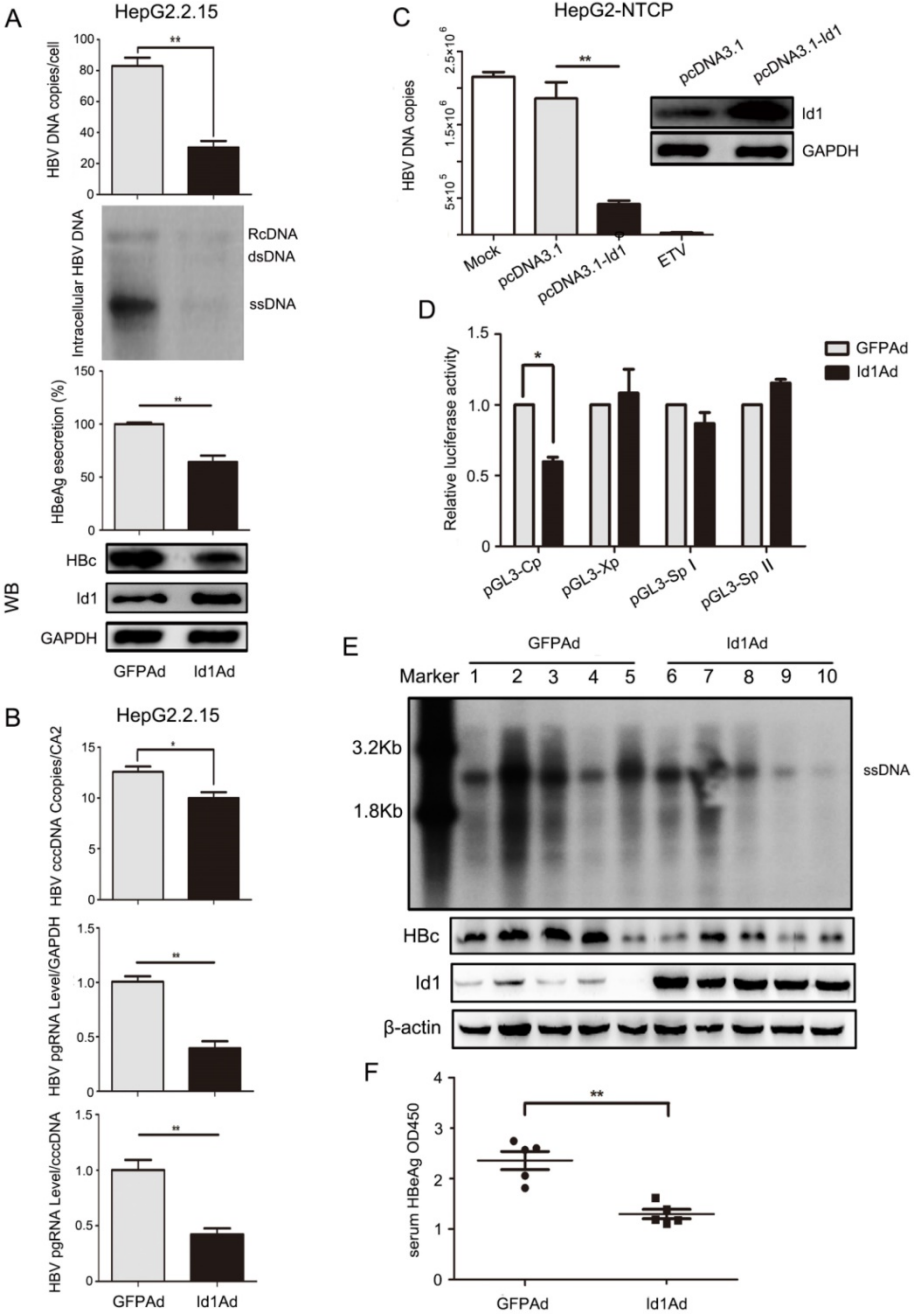
Enhancing expression of Id1 inhibited HBV transcription and replication *in vitro* and* in vivo*. **(A)** Overexpressing Id1 inhibited HBV DNA intermediates extracted from intracellular nucleocapsid 3 days after HepG2.2.15 cells infected with Id1 adeno-associated virus (Id1Ad). HBV DNA subsequently were subjected to qPCR and Southern blotting. The lysates from cells infected with Id1Ad were analyzed by western blotting with anti-Id1 and anti-HBc antibodies. GAPDH expression was used as loading control. HBeAg ELISA were used to screen culture supernatants 3 days after transfection. **P < 0.01. n = 5. **(B)** HBV covalently closed circle DNA (cccDNA) and pgRNA were subjected to PCR quantification using specific primers with CA2 and GAPDH as the reference gene, respectively. *P < 0.05 and **P < 0.01. n = 5. **(C)** HepG2-NTCP cells were firstly infected with 800 HBV viral particles/cell for 24 hours, and followed by transfection with pcDNA3.1-Id1 plasmid or pcDNA3.1 vector or medication with ETV. HBV DNA in cytoplasm was measured by qPCR after culture for additional 96 hours. All the qPCR data are presented as the mean±SE of triplicate experiments. **P < 0.01. n = 5. **(D)** Effect of Id1 overexpression on four HBV promoters. Different luciferase reporter vectors were co-transfected with Id1Ad or GFPAd into HepG2 cells. pRL-TK was co-transfected to normalise the transfection efficiency. The luciferase activity was measured at 48 h post-transfection. *P < 0.05. n = 5. **(E)** The mice were randomly allotted to two groups of 5 individuals per group. 7×10^9^ GFU Id1Ad (n1-n5) or GFPAd (n6-n10) were dissolved in 0.3 ml 0.9% normal saline and injected through the tail vein into mice. The mice were sacrificed at 20 days after injection, and the liver tissue and serum were collected. Intracellular HBV DNA extracted from 10 mg liver tissue was analyzed by southern blotting (top panel). The expression of Id1 and HBc in liver tissue lysates was tested by Western blotting with β-actin as an internal control (bottom panel).** (F)** The relative level of HBeAg in serum samples of AAV/HBV-infected mice were subjected to ELISA kits, **P <0.01. n = 5.

To detect the effect of Id1 on HBV regulation, Id1-expressing adenovirus (Id1Ad) was constructed and added to the HBV-expressing cell lines. Intracellular HBV DNA, HBc, and the extracellular HBeAg were less abundant in HepG2.2.15 cells infected with Id1Ad compared with those infected with GFP-expressing adenovirus (GFPAd) (Fig. 2A). A similar decline of HBV was also found in HepAD38 and HepG2-HBV1.1 cell lines with Id1 overexpression (Fig. S2A-B). Additionally, both the number of cccDNA copies and the abundance of pregenomic RNA (pgRNA), the reverse transcription template of HBV DNA, were reduced in HepG2.2.15 cells with Id1 upregulation (Fig. 2B); nevertheless, the reduction of pgRNA was much more drastic than that of cccDNA, suggesting that Id1 might regulate HBV via transcription, rather than directly affecting cccDNA abundance. A downward trend was detected in the accumulation of HBV DNA in HepG2-NTCP cells infected with HBV by transfecting pcDNA3.1-Id1 (Fig. 2C). It is well known that the transcription of HBV is mediated by four promoters. However, we found that only the activity of Cp was suppressed by Id1 (Fig. 2D), which is consistent with the decline of HBc and HBeAg.

Next, we investigated whether HBV was affected by Id1 *in vivo*. Compared to those of the control group, the levels of HBV DNA intermediates and HBc extracted from liver tissue of the experimental AAV/HBV-infected mice infected with Id1Ad were reduced to approximately 54% and 40%, respectively (Fig. 2E and S2C). In addition, HBeAg accumulation, which reflects the transcriptional activity of HBV *in vivo*, was also lower in the serum of mice treated with Id1Ad (Fig. 2F). Thus, these data confirmed that Id1 could reduce HBV transcription *in vitro* and *in vivo*.

### 3.3 E2F4 is a potential target gene of Id1 regulating HBV transcription

**Figure 3:**
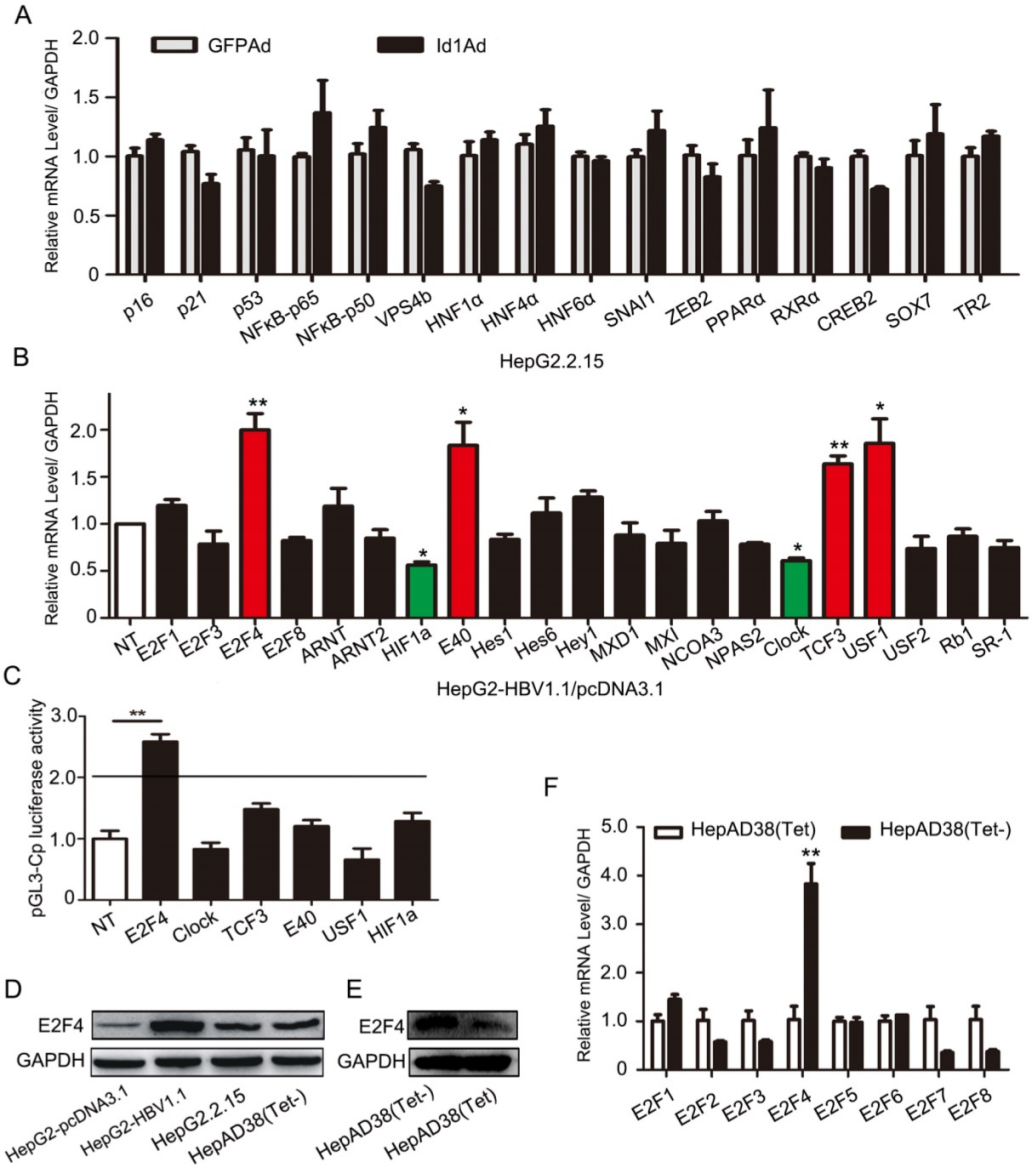
Regulation of HBV transcription was involved in E2F4. **(A)** Genes including P16, P21, p53, NFԟB-p65, NFԟB-p50, VPS4b, HNF1ɑ, HNF4ɑ, HNF6ɑ, SNAI1, ZEB2, PPARɑ, RXRɑ, CREB2, SOX7, and TR2, which have been reported to play important roles in transcription or replication of HBV, were screened. qPCR analysis of gene expression of various transcription factors related to HBV replication in HepG2.2.15 after being infected with Id1Ad. n = 3. **(B)** The qPCR analysis was subjected to screen gene expression of various HLH transcription factors possibly related to Id1 in HepG2 transfected with HBV1.1 plasmid. GAPDH expression was used as internal control. *P < 0.05 and **P < 0.01. n = 5. **(C)** Effects of a series of HLH transcription factors (including E2F4, CLOCK, TCF3, E40, USF1, HIF1ɑ ) overexpression on HBV Cp promoters were screened. Luciferase reporter vectors pGL3-Cp were cotransfected with overexpression plasmid into HepG2 cells. The cells were lysed and luciferase activity was determined at 48h after transfection. Meanwhile, the plasmid pRL-TK was used for normalizing the transfection efficiency. **P <0.01. n = 5. **(D)** The comparison of E2F4 protein level among HepG2, HepG2-HBV1.1, HepG2.2.15 and HepAD38 (without tetracycline). **(E)** The E2F4 protein level in HepAD38 cells with tetracycline-inhibiting HBV transcription was detected by western blotting with GAPDH used as the control gene. **(F)** The qPCR analysis was subjected to screen gene expression of various E2F transcription factor in HepAD38 cells with tetracycline-inhibiting HBV transcription. **P < 0.01. n = 5.

Previous experiments have shown that Id1 negatively regulated HBV transcription and replication *in vivo* and *in vitro*, however, the mechanism involved was completely unknown. For this, we screened 16 genes which have been reported to play roles in the transcription or replication of HBV and are potentially involved in viral replication, including P16, NFκB-P50/P65, HNF1α and HNF4α, et el. Nevertheless, there were few apparent differences in these transcription factor genes in HepG2.2.15 cells infected with Id1Ad (Fig. 3A), implying that Id1 might negatively regulate HBV through novel transcription factors. In line with the structural and functional characteristics of Id proteins, which can recruit E2F and bHLH proteins to form heterodimers, four *E2F* and 17 *bHLH* genes were subsequently detected in HepG2 cells transfected with HBV (Fig. 3B). The expression of four genes, namely *E2F4*, *E40*, *TCF3*, and *USF1*, significantly increased by about 2-fold, while that of Clock and *HIF1α* was reduced. Among these genes, only *E2F4* could dramatically improve luciferase activity driven by the core promoter by approximately 3-fold, whereas the other five exerted only slight effects (Fig. 3C). Moreover, a similar enhanced trend of E2F4 after HepG2-NTCP being infected with HBV was observed (Fig. S2D). Interestingly, the E2F4 protein level was notably higher in HBV-expressing HepG2-HBV1.1 and HepAD38 cell lines than in the respective control cell lines (Fig. 3D-E), whereas the other seven members of the E2F family did not show similar trends (Fig. 3F). According to Gene Expression Profiling Interactive Analysis (GEPIA), the expression levels of the two genes *E2F4* and *Id1* were also negatively correlated (Fig. S2E), implying a potential interplay between these two host factors in the regulation of HBV.

### 3.4 E2F4 positively regulates HBV transcription

**Figure 4:**
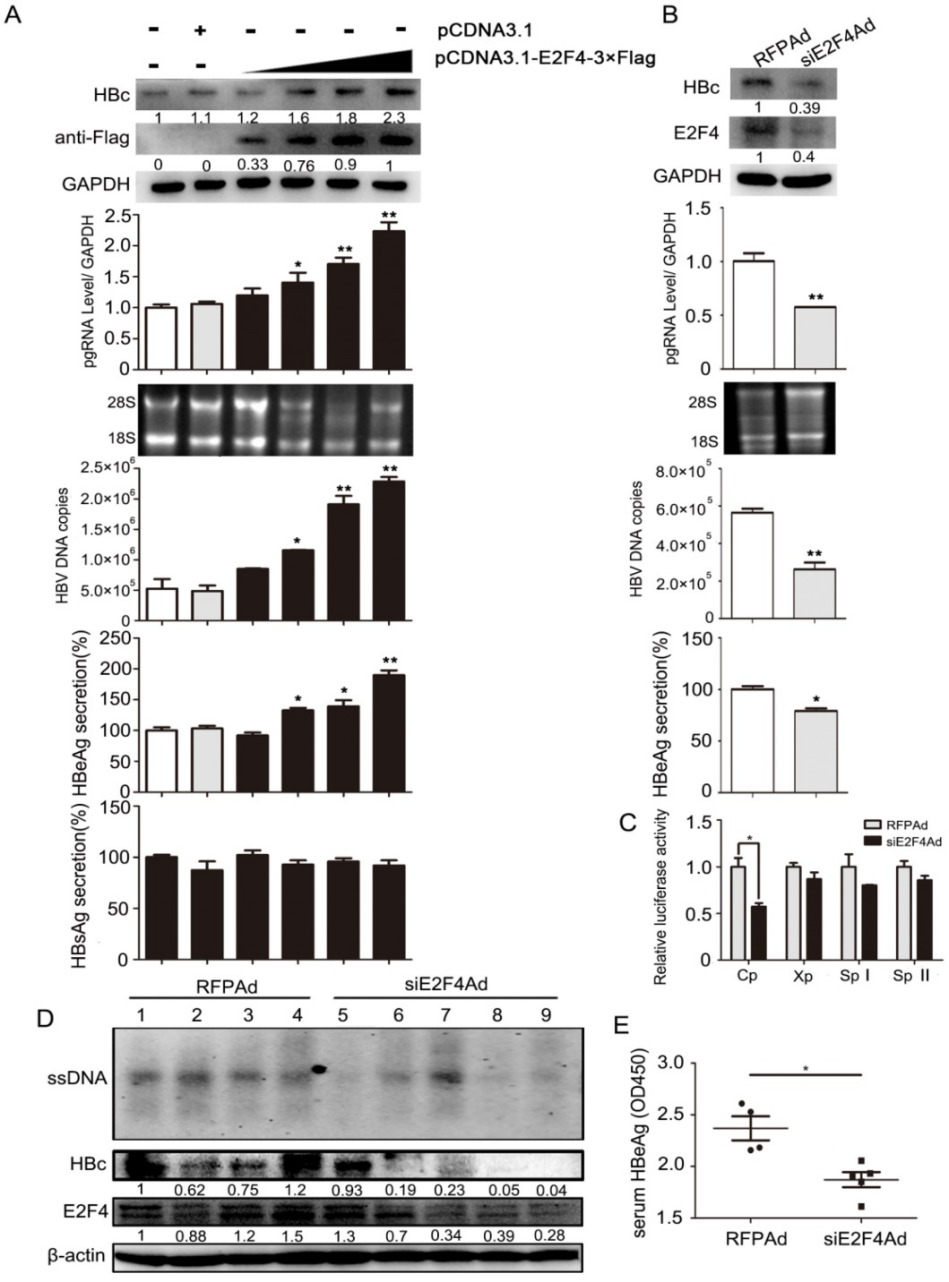
Regulation of HBV transcription *in vivo* and *in vitro* was positively mediated by E2F4. **(A)** HepG2.2.15 cells were transfected with an empty vector (pcDNA3.1) or increasing amounts of pcDNA3.1-E2F4-3×Flag expressing plasmids. After 3 days, expression of the Flag-tagged E2F4 and HBc proteins were analyzed by Western blotting. HBV DNA and pgRNA levels in cytoplasmic fractions were measured by qPCR, and expression of HBeAg and HBsAg in the supernatant of cell culture media were detected using ELISA. *P < 0.05 and **P < 0.01. n = 5. **(B)** HepG2.2.15 cells were infected with RFPAd or siE2F4Ad. After 3 days, E2F4 and HBc proteins, HBV DNA and pgRNA, and HBeAg were detected. *P < 0.05 and **P < 0.01. n = 5. **(C)** Effect of E2F4 on activity of HBV pGL3-Cp/Xp/SpI /SpII promoters were measured via dual luciferase reporter assay. *P < 0.05. n = 5. **(D)** The mice were randomly allotted to two groups of five individuals per group. Moderate siE2F4Ad or RFPAd were dissolved in 0.3 mL 0.9% normal saline and injected through the tail vein. The mice were sacrificed 20 days after injection and the liver tissue. Intracellular HBV DNA extracted from 10 mg liver tissue was analysed by Southern blotting (top panel). The expression of E2F4 and HBc in liver tissue lysates was tested by western blotting. β-actin protein level was used as an internal control.** (E)** The relative levels of HBeAg in serum samples were subjected by ELISA kits; **P < 0.01. n = 5.

To investigate whether E2F4 could regulate viral transcription, HepG2.2.15 cell lines were treated with E2F4 expression plasmids or siE2F4Ad (transcribing siRNA targeting E2F4). Results showed that the content of pgRNA, viral DNA, HBc, and HBeAg was markedly elevated in HepG2.215 with E2F4 overexpression, in a dose-dependent manner (Fig. 4A). Conversely, the levels of these four indicators markedly decreased in HepG2.215 cells infected with siE2F4Ad (Fig. 4B). Furthermore, a luciferase reporter assay, about HBV C, X, SPI, and SPII promoters induced by E2F4, showed that Cp activity decreased to approximately 55% in HepG2 cell lines infected with siE2F4Ad (Fig. 4C), consistent with the effect of Id1 on the same promoter activity (Fig. 2E). Additionally, we found that the level of HBV DNA intermediates and HBc in liver tissue and that of HBeAg in the serum from AAV/HBV-infected mice infected with siE2F4Ad was strikingly reduced compared to that in the control group (Fig. 4D and 4E).

### 3.5 E2F4 directly interacts with Id1 to regulate HBV

**Figure 5:**
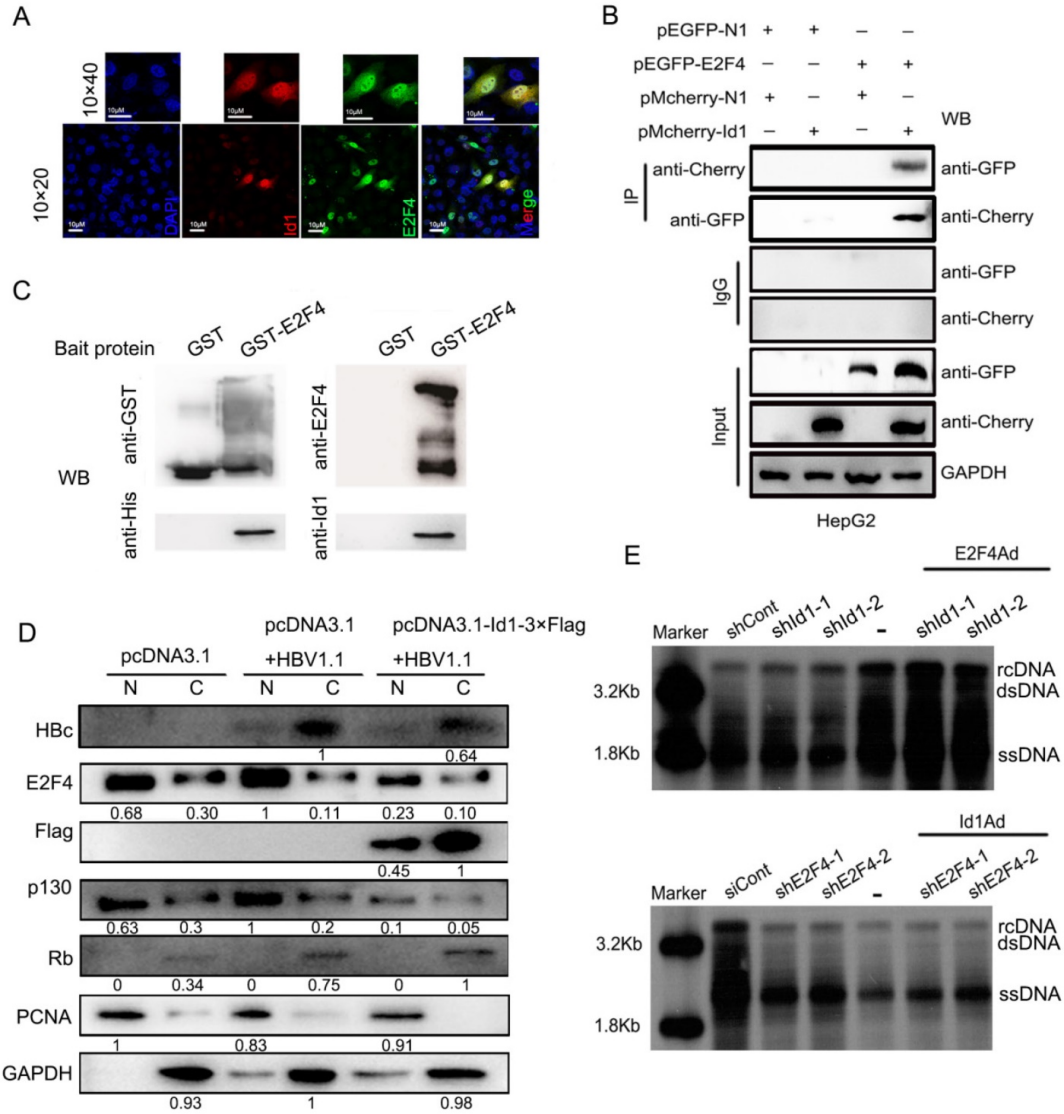
Transcription factor E2F4 was involved in Id1-mediated inhibition of HBV replication. **(A)** The location of Id1 (red) and E2F4 (green) in HepG2.215 cells under a natural state were detected by immunofluorescence images. DAPI (blue) were used to stain cell nucleus. Scale bar, 10 μm. **(B)** Lysates extracted from HepG2 cells expressing GFP-tagged E2F4 and Cherry-tagged Id1 were immunoprecipitated with anti-GFP or anti-Cherry, and the immunocomplexes were determined by immunoblot with respective anti-Cherry or anti-GFP antibody. **(C)** The western blot analysis following GST pull-down assay were performed with anti-Id1, anti-His, anti-E2F4, and anti-GST. The data showed that even though E2F4 expression in* E. coli* was unstable and easily degradable, Id1 also could be captured by E2F4.** (D)** Cytoplasmic-enriched(C) and nuclear-enriched(N) components from HepG2 cells (transfected with pCDNA3.1, pCDNA3.1+HBV1.1 and pcDNA3.1-Id1-Flag+HBV1.1, respectively) treated with a nuclear protein extraction kit were analyzed by immunoblot. Expression of GAPDH and PCNA, as markers for cytoplasm and nucleus respectively, was assessed by their specific monoclonal antibodies. The specificity of RB, p130, E2F4 and HBc bands were confirmed by their the specific antigenic peptides except Id1 by flag-antibody. **(E)** Southern blotting of HBV DNA was subjected to HepG2.2.15 cells after transfection early with shRNA-1/2 targeting Id1 and then infection with E2F4Ad. Southern blotting of HBV DNA was subjected to HepG2.2.15 cells after transfection early with shE2F4-1/2 targeting E2F4 and then infection with Id1Ad. The scramble control shRNA (shCont) was used as control group.

These results above implied that E2F4 could regulate HBV via transcription. The subcellular localization of Id1 and E2F4 by immunofluorescence revealed their binding potential in both the cytoplasm and the nucleus of HepG2.215 cells (Fig. 5A). When pmCherry-Id1 and pEGFP-E2F4 (enhanced GFP) fusion proteins were overexpressed in HepG2 cells, immunoprecipitation of Id1 by anti-mCherry antibody led to co-precipitation of E2F4 and vice versa (Fig. 5B). Moreover, pull-down assays were used to verify that Id1 could also be captured by E2F4* in vitro*; results demonstrated that Id1 directly interacts with E2F4 (Fig. 5C).

E2F4 can enter the nucleus with the help of pocket proteins. We observed that E2F4 was much more abundant in the nucleus, together with its interacting protein P130, in HepG2 transfected with HBV1.1 than control HepG2, and nuclear translocation of the two proteins was clearly blocked by overexpression of Id1 (Fig. 5D). To analyze the functional relationship between Id1 and E2F4, we infected HepG2.2.15 cells with E2F4Ad and shRNA against *Id1*, or with Id1Ad and shRNA against *E2F4*. Southern blot results showed that the intensification of HBV replication induced by silencing of *Id1* was further enhanced by E2F4 overexpression (Fig. 5E, top panel), whereas overexpression of Id1 did not dramatically facilitate the inhibitory effect of *E2F4* knockdown on HBV replication (Fig. 5E, bottom panel). These results further demonstrated that E2F4 is involved in Id1-mediated regulation of HBV replication.

### 3.6 E2F4 recognizes cccDNA at 1758'-TTAAAGGTC-1766' for positive regulation of HBV

**Figure 6:**
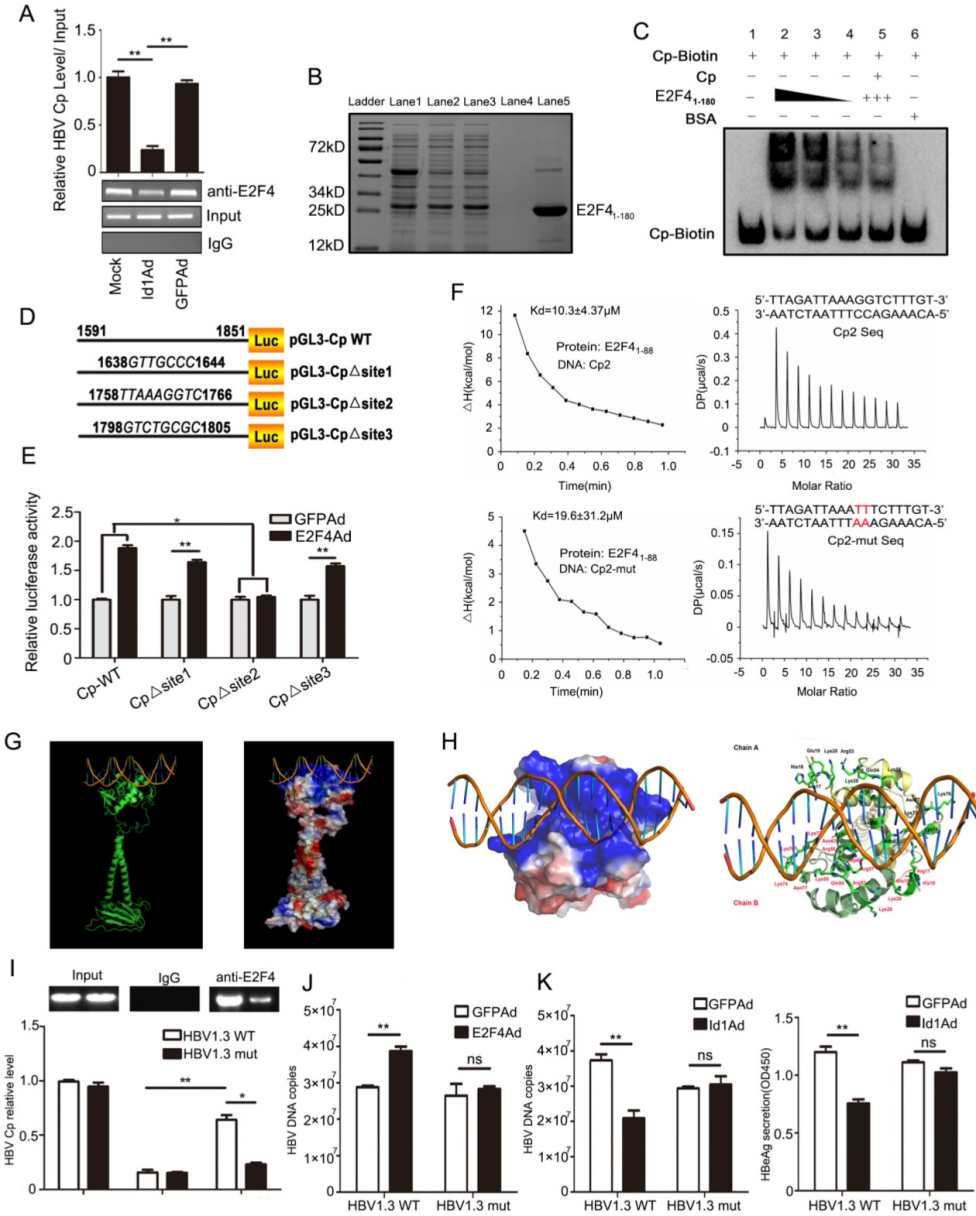
Mutated HBV Cp binding site of 1758-TTAAAGGTC-1766 abolished E2F4-induced promotion of HBV and Id1-induced inhibition of HBV replication. **(A)** ChIP assays were performed to confirm the interaction between E2F4 and HBV Cp. Lysates extracted from HepG2.2.15 were immunoprecipitated with anti-E2F4, and the immunocomplexes were subjected to PCR with specific primers. **P < 0.01. n = 3. **(B)** His-tagged pET28a-E2F4*_1-180_* constructs were expressed in *Escherichia coli* BL21 (DE3) cells induced with 0.2 mM IPTG. Truncated E2F4*_1-180_* protein were purified using Ni-NTA affinity column followed by stain with Coomassie Brilliant Blue dye. Ladder, protein molecular-mass markers; Lane 1, cell lysate after ultrasonication; Lane 2, cell supernatant after ultrasonication; Lane 3, Ni-NTA affinity column flow through fraction; Lane 4, eluted target protein with 20 mM imidazole; Lane 5, eluted target protein with 500 mM imidazole and followed with hyperfiltration. According the grayscale analysis by* Image J* software, the purity of E2F4*_1-180_* in the total eluted protein was higher than 90%. **(C)** The binding activity of E2F4*_1-180_* protein to the HBV Cp double-stranded probes amplificated by PCR presented in a dose-dependent mode, which determined by EMSA according to the manufacturer's protocol. Specificity of the DNA-protein complex was confirmed by competition with a 10-fold molar excess of unlabeled double-stranded DNA. BSA was used as negative control. **(D)** pGL3-Cp△site1, pGL3-Cp△site2 and pGL3-Cp△site3 were the deletion mutants of pGL3-Cp, whose deletion sites were respective nt1638-1644, nt1758-1766 and nt1798-1805. **(E)** pGL3-Cp△site2 resulted in E2F4 losing the ability to activating HBV Cp, which suggested nt1758-1766 should be the potential bound sites for E2F4. *P < 0.05 and **P < 0.01. n = 5. **(F)** Interaction of HBV Core promoter truncation with E2F4*_1-88_* protein by ITC binding analysis. **(G)** The three-dimensional model of homodimer E2F4-DNA complex developed through homology modelling. Cartoon and electrostatic potential surface representation of the overall structure of the complex. A saturated red color indicates Ø<-10 kiloteslas/e and a saturated blue indicates Ø>10 kiloteslas/e; T = 293 K. **(H)** The enlarged image of the key residues at interaction sites between E2F4 and DNA fragment. The binding site is strongly positive charged region, and the sticks represent the 32 positive residues to interaction with DNA, including Arg17, His18, Glu19, Lys20, Lys28, Arg53, Glu54, Lys55, Arg56, Arg57, Asn60, Asn63, Lys73, Lys74, Lys76, and Asn77 from Chain A and B of the E2F4 homodimer. **(I)** When compared with HBV1.3-WT plasmids, a evident decrease of HBV1.3-mut (containing mutated E2F4 binding site) Cp DNA fraction bound to E2F4 was proved by CHIP performed in lysates extracted from HepG2 cells transfected with HBV1.3-mut. *P < 0.05 and **P < 0.01. n = 3. **(J)** The capacity of E2F4 to enhancing HBV DNA copies in HepG2 cells transducted with HBV was weakened when the binding sites occurred mutant. **P <0.01 and ns means no significance. n = 5. **(K)** The HepG2 cells infected with Id1Ad were cotransfected with plasmids HBV1.3-WT or HBV1.3-mut. The negative effect of Id1 on HBV replication and transcription were also abolished by binding sites mutant of E2F4. **P <0.01 and ns means no significance. n = 5.

To further elucidate the mechanism underlying E2F4-dependent HBV regulation, we assessed whether E2F4 could positively regulate HBV via the Cp through a chromatin immunoprecipitation (ChIP) assay. Interestingly, the Cp of cccDNA could be precipitated by an anti-E2F4 antibody (Fig. 6A). In addition, the recruitment of E2F4 by Cp was significantly attenuated by overexpression of Id1 in HepG2.2.15 and HepAD38 (Fig. 6A, S3A, and S3B). E2F4_1-180_ of ~90% purity was prepared for studying its' interaction with Cp* in vitro* (Fig. 6B). Notably, E2F4_1-180_ retained about 80% activity in stimulating Cp (Fig. S3C). Consistently, the speed of electrophoretic mobility shift of full-length Cp probes was reduced by truncated E2F4_1-180_ in a dose-dependent manner (Fig. 6C, Lanes 1-4). On the other hand, a 10-fold molar excess of non-labelled Cp, competitively binding to the recombinant protein, effectively reduced the electrophoretic movement resistance of Cp-biotin caused by E2F4_1-180_ (Fig. 6C, Lanes 2 and 5), indicating that the interaction of E2F4 with the Cp of cccDNA was specific. Additionally, compared with control DNA, truncated Cp (biotin-5' Cp) could bind to E2F4_1-180_ with an about 2-fold stronger affinity, as revealed by surface plasmon resonance (SPR) detection (Kd = 87.8 nM vs. 135.8 nM; Fig. S3D), pointing at a direct interaction of E2F4 with Cp. Considering the E2F4 binding site motif, 5'-TTTSSCGC-3', pGL3-Cp△site1, pGL3-Cp△site2 and pGL3-Cp△site3 were constructed. Compared with the wild-type sequence, the truncated mutant Cp△site2 failed to be activated by E2F4 (Fig. 6D and 6E), suggesting that 1758'-TTAAAGGTC-1766' is the potential site of E2F4 binding.

Size exclusion chromatography confirmed that E2F4_1-88_ assembled into a homodimer in solution, indicating the DNA binding potential of E2F4 (Fig. S3E and S3F). Unsurprisingly, E2F4_1-88_ interacted with the aforementioned DNA fragment with an over 2-fold stronger affinity than with the Cp mutants (K_d_ = 10.3 ± 4.37 μM vs. 19.6 ± 31.2 μM) (Fig. 6F). Docking of the E2F4_1-180_ homodimer to DNA was performed in MOE and was further optimized for energy minimization (Fig. 6G). According to the modeling of its complex structure, 32 residues from Chain A and B of the E2F4 homodimer formed a strongly positively charged groove interacting with the promoter dsDNA sequence 1758'-TTAAAGGTC-1766' (Fig. 6H). Next, we constructed a site-directed variant (1758'-TTAAATTTC-1766') of Cp site 2 in pGEM-HBV1.3, which led to failed recruitment of E2F4 by Cp (Fig. S3G and 6I). Finally, Id1 and its interacting protein E2F4 greatly diminished the capacity to repress and improve HBV replication and transcription, respectively (Fig. 6J-K).

### 3.7 E2F4 and Id1 are expressed in HBV-associated HCC tissues and regulate multiple HBV genotypes in an opposite manner

**Figure 7:**
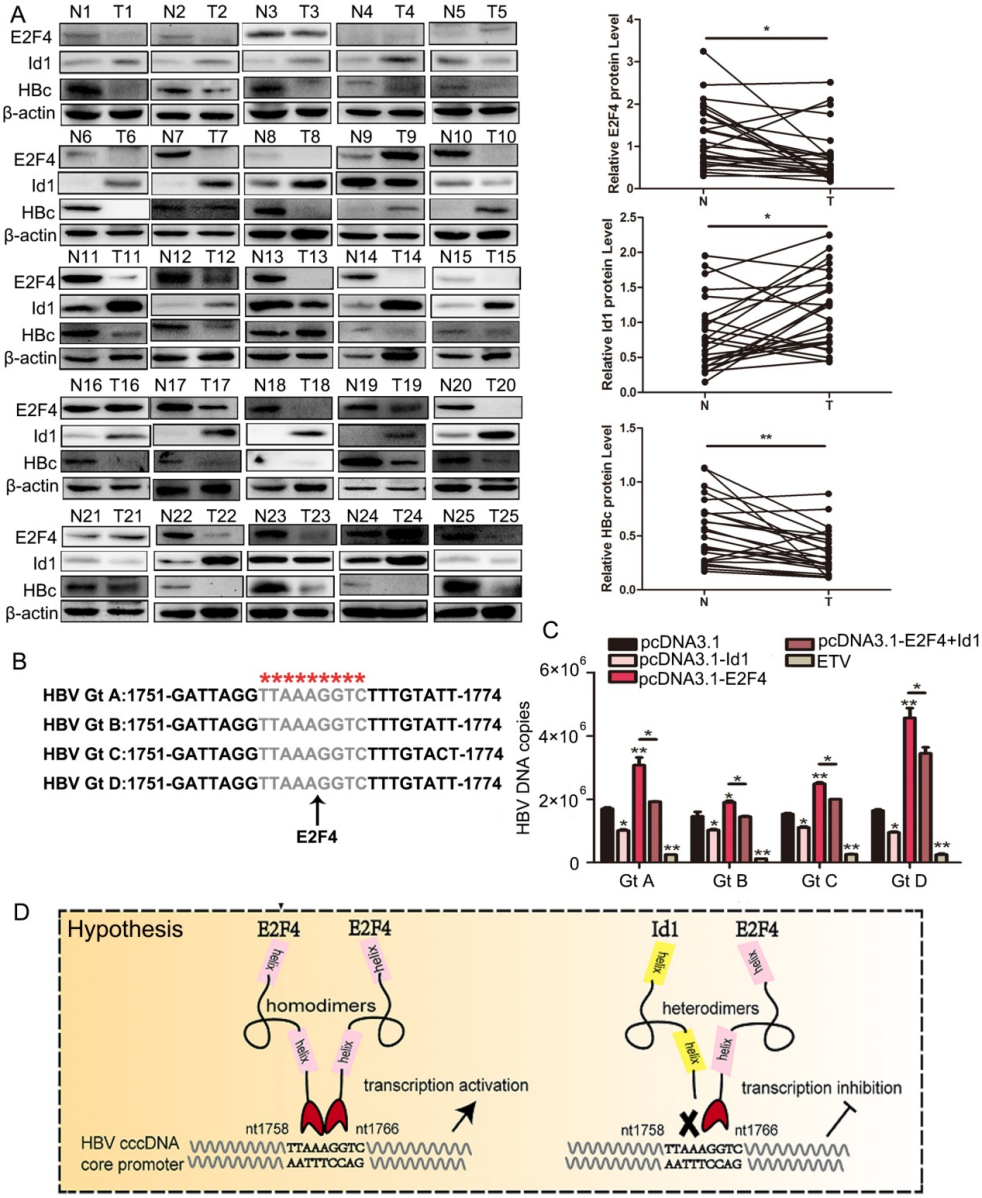
The comparison of Id1 and E2F4 expression in HBV-related HCC and schematic representation of the anti-HBV role of Id1. **(A)** The protein expression of E2F4, Id1 and HBc in TT and ANTT from 25 cases HBV-related HCC patient were tested by western blotting with β-actin used as the control protein. The right bar graph is the corresponding statistical figure. *P <0.05, **P < 0.01. n=25. **(B)** The conservatism of nt1758-1766 site sequence among four common HBV genotypes was shown. **(C)** HBV DNA were detected Id1 and E2F4 showed pan-genotypic antagonistic and synergistic effects on HBV respectively. *P < 0.05 and **P < 0.01. n = 5. **(D)** Schematic representation of E2F4-induced activation of HBV transcription and the anti-HBV role of Id1. NTCP, sodium taurocholate cotransporting polypeptide; rcDNA; relaxed circular DNA, cccDNA, covalently closed circular DNA; pgRNA, pregenomic RNA; ER, endoplasmic reticulum; Golgi, Golgi apparatus.

Next, we isolated tumor tissues (TT) and adjacent non-tumor tissues (ANTT) from 25 patients with HBV-associated HCC (Table S1) and compared their E2F4, Id1, and HBV expression. HBc protein levels were significantly higher in ANTT than in TT samples (in 20 out of 25 cases), and the mean level of HBV pgRNA was approximately 2-fold higher in ANTT than in TT (in 17 out of 25 cases) (Fig. 7A and S4A). Moreover, E2F4 expression was clearly higher in ANTT than in TT (in 17 out of 25 cases), whereas the mean level of Id1 protein was noticeably lower in ANTT than in TT (in 18 out of 25 cases). The clinical data attested that E2F4 could positively regulate HBV replication and transcription, but Id1 showed an opposite effect (Fig. 7A).

Additionally, 1758'-TTAAAGGTC-1766' is highly conserved among the four HBV dominant genotypes, namely A, B, C, and D (Fig. 7B). Oerexpressed Id1 and E2F4 could suppress and activate the replication of the four HBV1.3 genotypes, respectively (Fig. 7C and S4B). Altogether, our study demonstrated that the cccDNA binding protein E2F4 functions as an HBV transcription factor recognizing the Cp promoter at site 1758'-TTAAAGGTC-1766' and that Id1, blocking E2F4 recruitment, prevents E2F4-induced activation of HBV transcription (Fig. 7D).

## 4. Discussion

In this study, we first demonstrated that Id1 suppresses viral transcription and replication as well as the accumulation of pgRNA, viral proteins, HBV DNA, and cccDNA. As Id2, the homolog of Id1, can interact with E2F4 and E2F4 can activate HBV, we aimed to clarify the relationship between Id1 and E2F4 in HBV regulation 34. Our subcellular localization study showed that the Id1 and E2F4 proteins are located in both the cytoplasm and the nucleus, and immunoprecipitation and pull-down experiments further confirmed that Id1 and E2F4 directly interact with each other.

cccDNA serves as a template for the replication of HBV DNA genomes and transcription of viral RNAs. Consequently, silencing cccDNA is a major tactic to reduce HBV virulence. Recently, several strategies with less hepatotoxicity targeting cccDNA have been reported. For instance, a CRISPR/Cas9 system or the overexpression of Smc5/6 complex and protein arginine methyltransferase 1 (PRMT1) could suppress cccDNA transcription or replication through different mechanisms 35,36. E2F4, a novel cccDNA-binding protein, can form heterodimers with other bHLH proteins, such as Id2, P130, Dp and pRB 37. In this work, we revealed that E2F4 could recognize the Cp region 1758'-TTAAAGGTC-1766' with strong specific affinity. Consequently, E2F4 might be a novel target for the inhibition of HBV infection. Maybe, this recognization progress need more transcription factors, such as DP-1, which required further study on interaction among E2F4, DP-1 and Id1.

Viral and host proteins that either directly bind to cccDNA sequences or to the minichromosome by protein-protein interactions have become a research hot spot in both virology and pharmacology. Currently, HBc and HBx, H3 and H4 histones, cellular transcription factors, and chromatin modification enzymes have been shown to be associated with cccDNA by ChIP assays 36. According to our experiments, E2F4 was up-regulated in ANTT carrying high HBV loads with compared to TT, which is consistent with previous results reported by Li et, al., could directly interact with the cccDNA Cp region 1758'-TTAAAGGTC-1766' ; Moreover, the host factors SP1, HNF4 and PPAR can also directly bind to cccDNA at the adjacent locus 1745-1775 38. Such a limited region could be saturated upon binding of only one or two proteins. Virus in hepatitis B-related HCC patients are more likely to occurring A1762T/G1764A Cp mutants, which influences the expression of both the pre-core/core and the pregenomic (pg) RNA transcripts and lead to Cp-mutant might not be recognized by E2F4 and explain why low expression of HBV in TT. More and more academics regard the two mutants as strong predictors of HCC risk 39-41. Therefore, future studies on the assembly of the chromosomal structure of HBV involved in Id1, E2F4, etc. host proteins and epigenetic modification of cccDNA, across the different stages of HBV transcription, replication, or quiescence are required.

In addition, our results provide a possible explanation to why expression of HBV DNA and viral proteins was significantly lower in clinical HCC tissues than in ANTT, a phenomenon that has attracted increasing attention in recent years 15,42. Among the known regulatory proteins of HBV, HNF6α, p16, and p53 negatively regulate HBV replication and exhibit much lower expression in HCC tissues than in ANTT. Similarly, Id1 could negatively regulate HBV but was more strongly expressed in HCC tissues than in ANTT; this may represent another key factor triggering decreased HBV replication and transcription in HCC tissues. We speculate that once Id1 levels decrease, the Id1-E2F4 heterodimer dissociates; subsequently, E2F4 would regain the ability to bind cccDNA and promote the replication and expression of HBV. This mechanism may explain high HBV expression in ANTT.

In summary, for the first time, that E2F4 can form a homodimer to bind to the Cp region (1758'-TTAAAGGTC-1766') of HBV cccDNA and positively regulate HBV transcription. Finally, the high conservation of this E2F4 binding site on cccDNA among the four HBV genotypes pointed at E2F4 as a putative target for anti-HBV therapy. Further studies generating inhibitors based on the Id1 protein structure or targeting the interaction between E2F4 and cccDNA are crucial for the development of new strategies for antiviral therapy.

## Supplementary Material

Supplementary figures and tables.

## Abbreviations

HBV: hepatitis B virus
cccDNA: covalently closed circular DNA
rcDNA: relaxed circular DNA
dsDNA: double-stranded DNA
ssDNA: single-stranded DNA
Id1: Inhibitor of differentiation/DNA binding 1
E2F4: E2F transcription factor 4
NTCP: Na+/taurocholate cotransporting polypeptide
ChIP: chromatin immunoprecipitation
HCC: hepatocellular carcinoma
GAPDH: glyceraldehyde-3-phosphate dehydrogenase
HBc: hepatitis B viral core protein
Cp: HBV core promoter
Id1Ad: Id1 adenovirus
GFPAd: GFP adenovirus
HLH: helix-loop-helix protein family
HBeAg: Hepatitis B e Antigen
HBsAg: Hepatitis B s Antigen
TT: tumor tissue
ANTT: adjacent non-tumor tissue
EMSA: Electrophoretic Mobility Shift Assay
SPR: surface Plasmon resonance
RB: retinoblastoma susceptibility gene 1 product
