# Supplementary material for: Cellular Id1 inhibits hepatitis B virus transcription by interacting with the novel covalently closed circular DNA-binding protein E2F4
Source: Int J Biol Sci. 2022 Jan 1;18(1):65–81. doi: 10.7150/ijbs.62106 (PMC8692152; doi:10.7150/ijbs.62106)
Supplement: Supplementary file 1 — Supplementary figures and tables. [file ijbsv18p0065s1.pdf]

Cellular Id1 inhibits hepatitis B virus transcription by interacting with the novel covalently closed circular DNA-binding protein E2F4

Jie Wei[1,3¶], Yueyuan Shi[2,5¶], Chunhong Zou[2], Hongpeng Zhang[6], Hui Peng[4], Shilei Wang[2], Lulu Xia[2], Yuan Yang[2], Xiang Zhang[2] Junye Liu[2], Hua Zhou[7], Miao Luo[5], Ailong Huang[1,*], Deqiang Wang[1,2,*]

Supplementary Table

Table S1. Patient characteristics (N = 25)

Age (years)*	53.4（22-77）
Sex (female/male)	4/21
HBsAg (-/+)*	7/18
HBV DNA (</>1.0*$10e^3$ copies/ml)*	11/14
AST (IU/L)*	156.88(24-1608)
ALT (IU/L)*	135.46(16-1491)
AFP (ng/ml) *	18494.5(0-242611)
Cirrhosis (presence/absence)**	15/10

Table S2. Primer sequences used for clone and site-specific mutagenesis

Gene	Primer Sequence (5'→3')
Id1Ad	Sense:CG*GGATCC*ATGAAAGTCGCCAGTGGCAGC<br>Anti-sense:CCC*AAGCTT*TCAGCGACACAAGATGCGATCG
pcDNA3.1-Id1	Sense:TTA*GGATCC*ATGAAAGTCGCCAGTGGCAGC<br>Anti-sense:ATT*CTCGAG*TCAGCGACACAAGATGCGATCG
E2F4Ad/<br>pcDNA3.1-E2F4	Sense: ATT*GGATCC*ATGGCGGAGGCCGGGCCACAG<br>Anti-sense:TCC*CTCGAG*TCAGAGGTTGAGAACAGGCAC
pcDNA3.1-CLO<br>CK	Sense:ATT*GCTAGCA*TGTTGTTTACCGTAAGCTG<br>Anti-sense:TCC*CTCGAG*CTACTGTGGTTGAACCTTGG
pcDNA3.1-TCF3	Sense:ATT*GGATCC*ATGAACCAGCCGCAGAGGAT<br>Anti-sense:TCC*TCTAGA*TCACATGTGCCCGGCGGGGT
pcDNA3.1-E40	Sense:TTA*GAATTC*ATGGAGCGGATCCCCAGCGC<br>Anti-sense:TGC*TCTAGA*TTAGTCTTTGGTTTCTAAG
pcDNA3.1-USF1	Sense:ATC*GGATCC*ATGTACAGGGTGATCCAGGTG<br>Anti-sense:TGC*TCTAGA*TTAGTTGCTGTCATTCTTG
pGEX-E2F4(GS<br>T-E2F4)	Sense:ATT*GGATCC*ATGGCGGAGGCCGGGCCACAG<br>Anti-sense:TCC*CTCGAG*TCAGAGGTTGAGAACAGGCAC
pET-Id1(Id1-His)	Sense:CG*GGATCC*ATGAAAGTCGCCAGTGGCAGC<br>Anti-sense:CCC*AAGCTT*TCAGCGACACAAGATGCGATCG
pEGFP-E2F4	Sense:TCAGATCTCGAGCTCAAGCTTGCCACCATGGCGGA<br>GGCCGGGCCACAG<br>Anti-sense:CATGGTGGCGACCGGTGGATCCCGGAGGTTGA<br>GAACAGGCAC

pMcherry-Id1	Sense:TCAGATCTCGAGCTCAAGCTTGCCACCATGAAAGTCGCCAGTGGCAGC<br>Anti-sense:CATGGTGGCGACCGGTGGATCCCGGCGACACAAGATGCGATCG
pET-E2F4$_{1-180}$	Sense:ATT*GGATCC*ATGGCGGAGGCCGGGCCACAG<br>Anti-sense:TCC*AAGCTT*CTGGTACTTCTTCTGCCC
pcDNA3.1-E2F4$_{1-180}$	Sense:ATT*GGATCC*ATGGCGGAGGCCGGGCCACAG<br>Anti-sense:TCC*CTCGAG*CTGGTACTTCTTCTGCCC
pGL3-Cp△site1	Sense:GAACGCCCACCGAATAAGGTCTTACATAAG<br>Anti-sense:CTTATGTAAGACCTTATTCGGTGGGCGTTC
pGL3-Cp△site2	Sense:GGAGGAGATTAGATTTGTACTAGGAGGCTG<br>Anti-sense:CAGCCTCCTAGTACAAATCTAATCTCCTCC
pGL3-Cp△site3	Sense:TGTAGGCATAAATTGACCAGCACCATGCAAC<br>Anti-sense:GTTGCATGGTGCTGGTCAATTTATGCCTACA
HBV1.3 mut	Sense:GGAGATTAGATTAAATTTCTTTGTACTAGGAG<br>Anti-sense:CTCCTAGTACAAAGAAATTTAATCTAATCTCC
Cp(for EMSA)	Sense: TCACCTCTGCACGTCGCATG<br>Anti-sense:AGTTGCATGGTGCTGGTGCG
Biotin-Cp(for EMSA)	Sense:5'-Biotin-TCACCTCTGCACGTCGCATG<br>Anti-sense:AGTTGCATGGTGCTGGTGCG
Control-DNA-biotin(for SPR)	Sense:5'-Biotin-ATGATGTCACCACAAGAAGT<br>Anti-sense:ACTTCTTGTGGTGACATCAT
Biotin-Cp2(for SPR)	Sense:5'-Biotin-TTAGATTAAAGGTCTTTGT<br>Anti-sense:ACAAAGACCTTTAATCTAA
Cp2(for ITC)	Sense:TTAGATTAAAGGTCTTTGT<br>Anti-sense:ACAAAGACCTTTAATCTAA
Cp2-mut(for ITC)	Sense:TTAGATTAAATTTCTTTGT<br>Anti-sense:ACAAAGAAATTTAATCTAA

Table S3. Target sequences of shRNAs.

Name of shRNAs	Target sequence (5’-3’)
shId1-1	Sense:GGCGAGATCAGCGCCCTGA<br>Anti-sense:TCAGGGCGCTGATCTCGCC
shId1-2	Sense:CGGCATGCGTTCCTGCGGA<br>Anti-sense:TCCGCAGGAACGCATGCCG
shE2F4-1	Sense:CGGCGGATTTACGACATTA<br>Anti-sense:TAATGTCGTAAATCCGCCG
shE2F4-2	Sense:CTCACGTCCAAATAGTCCT<br>Anti-sense:AGGACTATTTGGACGTGAG
siE2F4Ad	Sense:CGGCGGATTTACGACATTA<br>Anti-sense:TAATGTCGTAAATCCGCCG

Table S4. Primer sequences used for qPCR.

Gene	Primer Sequence (5’→3’)

Id1	Sense:TGTTACTCACGCCTCAAGG<br>Anti-sense:AACTGAAGGTCCCTGATGTAG
p16(CDKN2A)	Sense:ACCAGAGGCAGTAACCATGC<br>Anti-sense:AAGTTTCCCGAGGTTTCTCAG
p21(CDKN1A)	Sense:TGTCCGTCAGAACCCATGC<br>Anti-sense:AAAGTCGAAGTTCCATCGCTC
p53(TP53)	Sense:GGAAATTTGCGTGTGGAGTATTT<br>Anti-sense:GTTGTAGTGGATGGTGGTACAG
NFκB-P50	Sense:CAAGGACATGGTGGTCGGCTTC<br>Anti-sense:CGCCTCTGTCATTCGTGCTTCC
NFκB-P65	Sense:TGATAGCGACTCGGACTCTGAAGG<br>Anti-sense:CAAGTGAGGTCAAGAGGCGTGTG
E2F1	Sense:GCTGGACCACCTGATGAATATC<br>Anti-sense:GTCTGCAATGCTACGAAGGT
E2F3	Sense:TGTCCAGAAGAGACTGAAACAC<br>Anti-sense:GCTATGTCCTGAGTTGGTTGAAG
E2F5	Sense:GGCTGCTCACTACCAAGTTC<br>Anti-sense:TGCCTCACAGCCAAAGTATC
E2F8	Sense:GTGTCAACGCCTCAGATAGTAAG<br>Anti-sense:GATCCAGGCTACTCAGAACATTAG
ZEB2	Sense:TTTCAGGGAGAATTGCTTGA<br>Anti-sense:CACATGCATACATGCCACTC
SNAIL1	Sense:CTCTTTCCTCGTCAGGAAGC<br>Anti-sense:GCTGGAAGGTAAACTCTGGATTA
HNF6a	Sense:CACAGATGTCCAGCGTCGAACTC<br>Anti-sense:TCCTGCCACTTGTCCAGACTCC
PPARa	Sense:TCCTCGGTGACTTATCCTGT<br>Anti-sense:GCGTGGACTCCGTAATGATAG
RXRa	Sense:CATCGTCCTCTTTAACCCTGAC<br>Anti-sense:GGGTACTTGTGCTTGCAGTA
TR2(NR2C1)	Sense:CCTCCAGACTGCTGTTCTTATC<br>Anti-sense:CTGGGCAAGACCAAGAGTAAA
CREB2(ATF4)	Sense:GGAGATAGGAAGCCAGACTACA<br>Anti-sense:GGCTCATACAGATGCCACTATC
SOX7	Sense:TCAGCAAGATGCTGGGAAAG<br>Anti-sense:CGGCCGGTACTTGTAGTTG
HNF4ɑ	Sense:CCTACCTCAAAGCCATCATCTT<br>Anti-sense:GTCGTTGATGTAGTCCTCCAAG
VPS4b	Sense:GCACTGAAAGAGGCTGTGATA<br>Anti-sense:CCAGGCGGCCCAAATAATA
ARNT	Sense:CTAAGAGACAGCTTCCAACAGG<br>Anti-sense:GTTCTCATCCAGAGCCATTCTT
ARNT2	Sense:TTTGGACCACCTTCCTCTAAAC<br>Anti-sense:GTACAGTGGACCACAGCATATT

HIF1α	Sense:GTCTGCAACATGGAAGGTATTG<br>Anti-sense:GCAGGTCATAGGTGGTTTCT
E40(BHLHE40)	Sense:GACAGCAAGGAGACCTACAAAT<br>Anti-sense:GTTTGAGATGTTCGGGTAGGAG
Hes1	Sense:CGACACCGGATAAACCAAAG<br>Anti-sense:CGCGAGCTATCTTTCTTCAGAG
Hes6	Sense:GACCTGTGCTCCGACCTG<br>Anti-sense:CAGACACTCCGGGCAATTTG
Hey1	Sense:CGACGAGACCGGATCAATAAC<br>Anti-sense:GCAGATCCCTGCTTCTCAA
MXD1	Sense:GACTCCGACAGGGAAGAAATC<br>Anti-sense:GCTGGAATAGCCCTCATCAC
MXL	Sense:GATTCAGAGCGAGAGGAGATTG<br>Anti-sense:CACTGATGCTGGTGGTACTTAT
NOCA3	Sense:CGGCAGAATGGAACCTATGAA<br>Anti-sense:GAAGAGGCAATGTGGGAATAGAG
NPAS2	Sense:CACAGAGCACCTCCAATCATAG<br>Anti-sense:CTCCAGGTCATCAATGTGGTAG
Clock	Sense:CTCTACTCATCTGCTGGAAAGTG<br>Anti-sense:ATGGCTCCTTTGGGTCTATTG
TCF3	Sense:CCCGGATCACTCAAGCAATAAC<br>Anti-sense:GTCGTAGCTGGGCGATAAGGC
USF1	Sense:CAGGGCTCAGCATAATGAAGT<br>Anti-sense:TCCATAGAGCAGTCTGGGATT
USF2	Sense:CGTCCAGTGTGGGAGATACT<br>Anti-sense:TGTTCCTGTCTGAAGCACATC
Rb1	Sense:CTGTCTGAGCACCCAGAATTAG<br>Anti-sense:GTCCAAATGCCTGTCTCTCAT
SR-1(SREBF1)	Sense:CACTGAGGCAAAGCTGAATAAAT<br>Anti-sense:TAGGTTCTCCTGCTTGAGTTTC
pgRNA	Sense:GCCTTAGAGTCTCCTGAGCA<br>Anti-sense:GAGGGAGTTCTTCTTCTAGG
HBV DNA	Sense:TGCGGCGTTTTATCATATTCC<br>Anti-sense:ATACCTTGGTAGTCCAGAAGAACCA
HBV cccDNA	Sense:GTGCACTTCGCTTCACCTCT<br>Anti-sense:AGCTTGGAGGCTTGAACAGT
GAPDH	Sense:GTATGACAACGAATTTGGCTACAG<br>Anti-sense:TGAGGGTCTCTCTCTTCCTCTTGT
β-actin	Sense:CCTGGCACCCAGCACAAT<br>Anti-sense:GCCGATCCACACGGAGTA
CA2(Cyclin A2)	Sense:TTATTGCTGGAGCTGCCTTT<br>Anti-sense:CTCTGGTGGGTTGAGGAGAG
Cp(for CHIP)	Sense: TCACCTCTGCACGTCGCATG<br>Anti-sense:AGTTGCATGGTGCTGGTGCG

Table S5. Sequence of E2F4$_{1\text{-}88}$

ATGGCGGAAGCGGGCCCGCAGGCGCCGCCGCCGGGCACCCCGAGCC<br>GTCACGAAAAAAGCCTGGGCCTGCTGACCACCAAATTCGTTAGCCTGCTG<br>CAGGAAGCGAAAGATGGCGTTCTGGATCTGAAACTGGCGGCGGATACCCT<br>GGCGGTTCGTCAGAAACGTCGTATCTACGATATCACCAACGTTCTGGAAGG<br>CATCGGTCTGATCGAAAAGAAAAGCAAAAACAGCATCCAGTGGAAAGGCG<br>TTGGTCCGGGCTGC

Supplementary figures

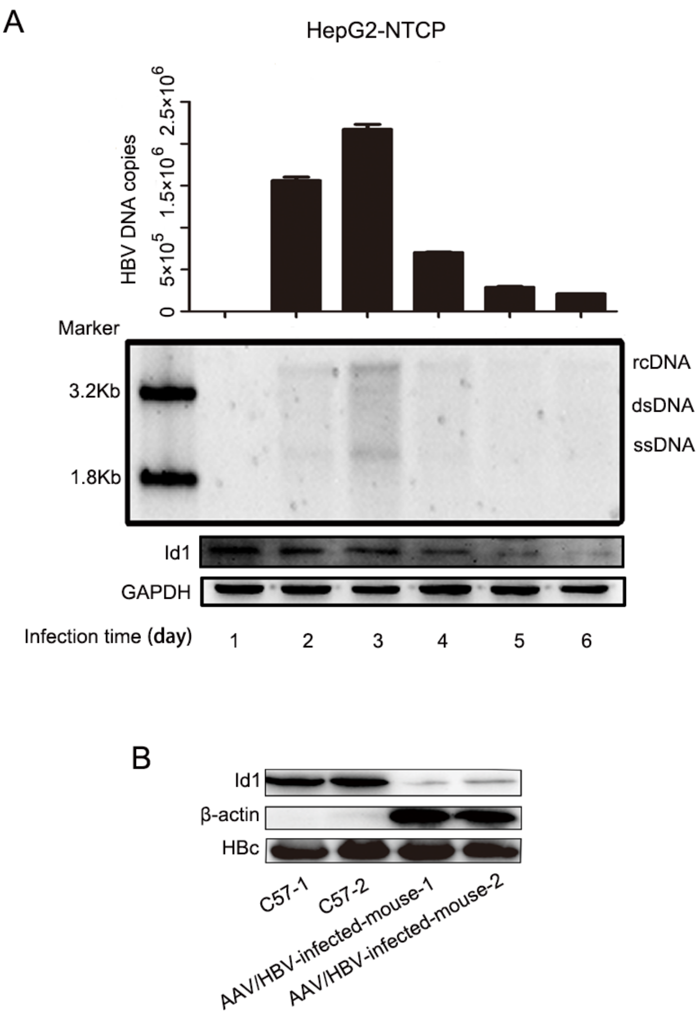

Figure S1: (A) 3.5×10^5 Cells infected with 800 HBV particles/cell were subjected to qPCR, southern blotting and western blotting at 1-6 day s post infection. In all conditions, 4% PEG8000 was added in the inoculum for promoting HBV infection, then washed out 24 hours post-infection. **(B)** Expression of Id1 in liver of AAV/HBV-infected mice. Livers were extracted from C57 black/6J (no HBV replication) or AAV/HBV-infected mice (HBV replication) and subjected to Western blotting with β-actin used as the control gene.

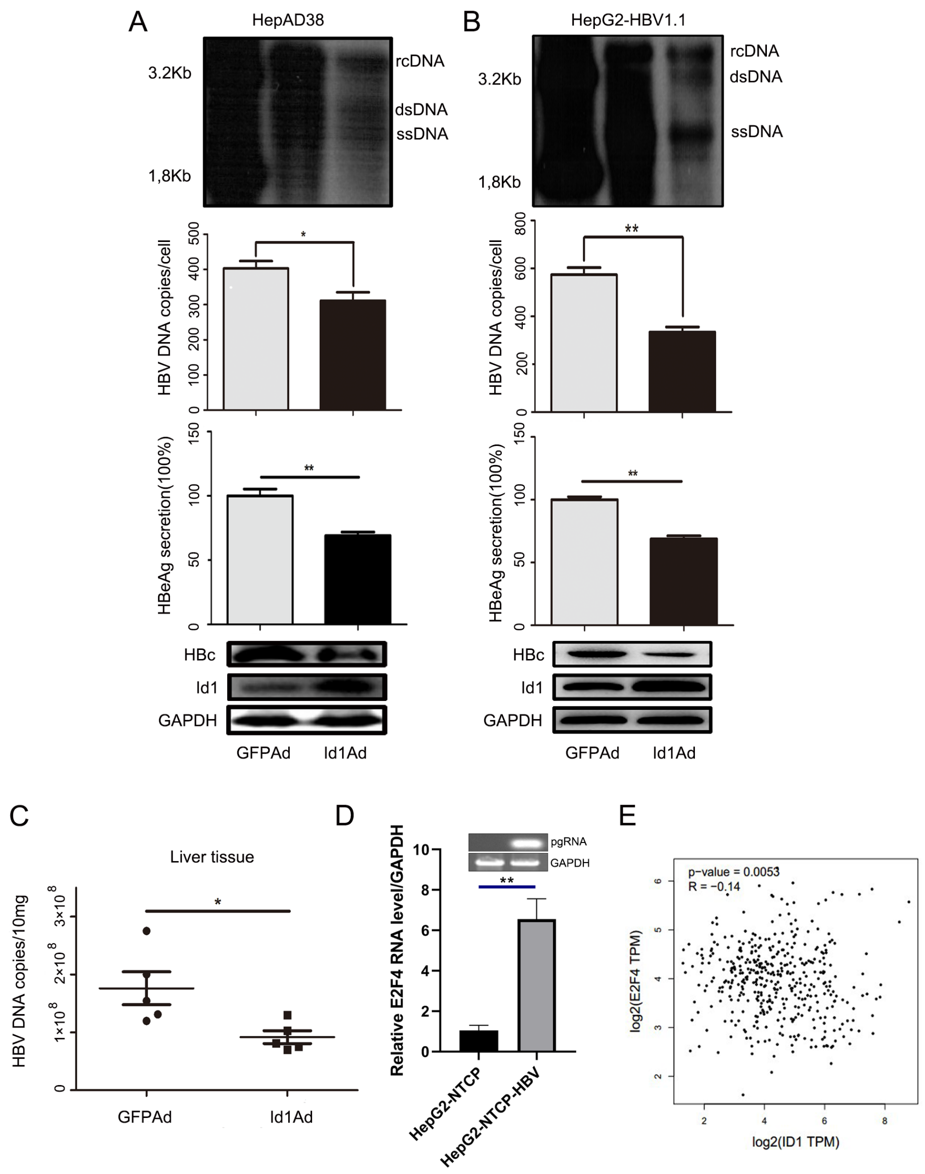

Figure S2: (A-B) Detection of inhibition of Id1 on HBV transcription and replication in HepAD38 and HepG2-HBV1.1 cells. Intracellular HBV DNA was extracted and subjected to qPCR and Southern blotting 72 h after addition of Id1Ad to the cells. HBeAg and HBc protein were detected by ELISA and western blotting, respectively. GAPDH was used as a loading control. GFPAd was used as control adenovirus. **(C)** Intracellular HBV DNA extracted from 10 mg liver tissue of AAV/HBV-infected mice was analyzed by absolute quantification PCR. **(D)** Enhancement of E2F4 mRNA when HepG2-NTCP being infected with HBV was detected. **(E)** The correlation coefficient between E2F4 and Id1 in LIHC tumour and normal from GEPIA database was visualized by Spearman Analysis.

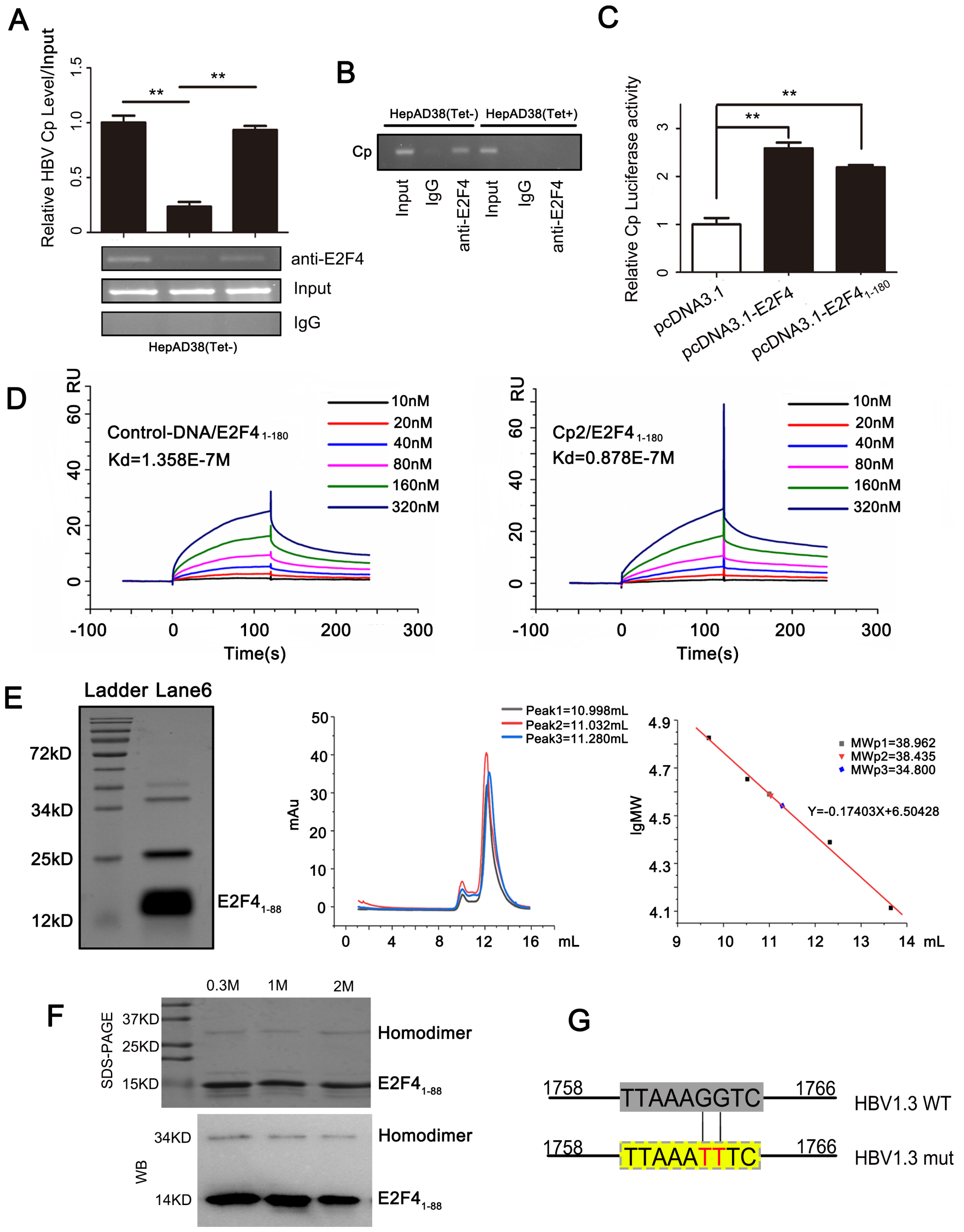

Figure S3: (A) ChIP assays were performed to confirm the interaction between E2F4 and HBV Cp in HepAD38 cells. **(B)** ChIP assays were performed to confirm the interaction between E2F4 and HBV Cp in HepAD38 cells with or without tetracycline. **(C)** Increase in the luciferase activity of Cp induced by E2F4*1-180* was slightly lower than E2F4 full-length protein, which revealed that E2F4*1-180* retained the ability to recognize and bind Cp DNA fraction. **(D)** Interaction of HBV core promoter with truncated E2F4*1-180* protein by SPR-binding analysis. E2F4*1-180* protein was injected

over a sensor chip immobilised with biotinylated-Cp DNA (bottom) or biotinylated-control DNA (top) as random control, and real-time binding profiles for increasing concentrations of $E2F4_{1\text{-}180}$ protein (black: 10 mM, red: 20 mM, light blue: 40 mM, pink: 80 mM, green: 160 mM, dark blue: 320 mM) were assessed. **(E-F)** Truncated $E2F4_{1\text{-}88}$ protein was purified. The purity of $E2F4_{1\text{-}88}$ in the total elution protein was higher than 80%. Elution Profile of $E2F4_{1\text{-}88}$ protein. The protein was separated and eluted with 50 mM Tris-HCl pH8.0 and 0.3M/1M/2M NaCl on a Superdex 75 gel filtration column. **(G)** The graphical representation of nt1758-1766 of HBV1.3 wild-type and mutant plasmid.

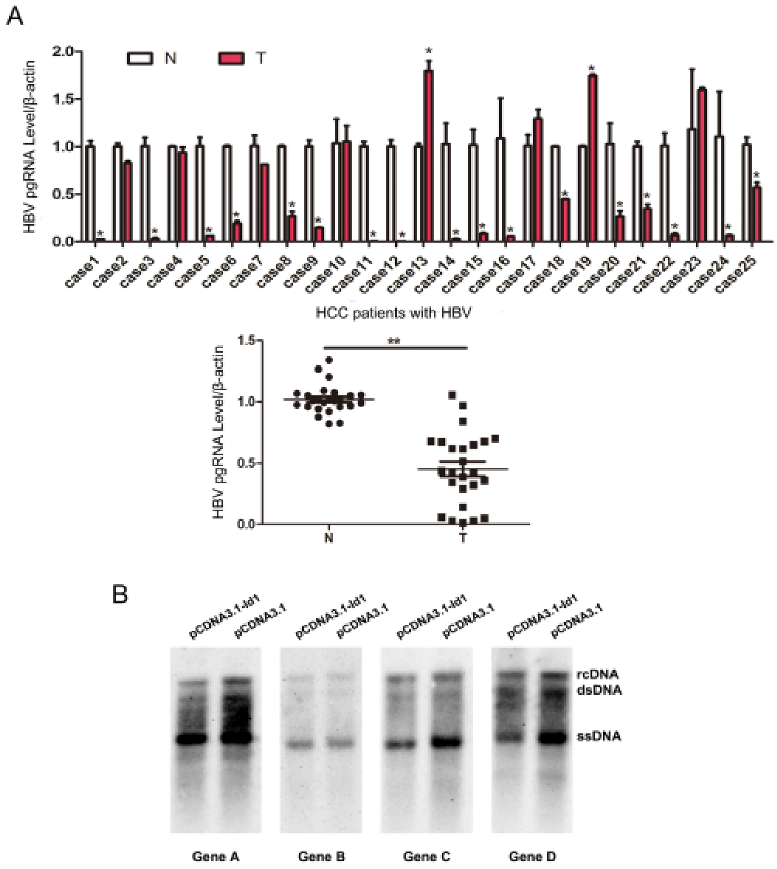

Figure S4: (A) The level of HBV pgRNA in tumor tissue (T) and adjacent non-tumor tissue (N) from 25 cases HBV-related HCC patients were tested by qPCR with β-actin used as the control gene. The bottom bar graph is the corresponding statistical figure. Data are presented as the mean±SE of triplicate experiments. **P* < 0.05, ***P* < 0.01. **(B)** Detection of suppression of pcDNA3.1-Id1 on HBV1.1 genotype A/B/C/D by Southern blotting with pcDNA3.1(+) vector used as control plasmid (NC).
